# Admissible dissimilarity value (ADV) as a measure of subsampling reliability: case study North Sea cod (*Gadus morhua*)

**DOI:** 10.1007/s10661-020-08668-6

**Published:** 2020-11-12

**Authors:** Julia Wischnewski, Matthias Bernreuther, Alexander Kempf

**Affiliations:** Johann Heinrich von Thünen Institute of Sea Fisheries, Herwigstraße 31, Bremerhaven, 27572 Germany

**Keywords:** Length frequency distribution Reference subsample Admissible dissimilarity measure, Robust distributional modes and antimodes, Sampling effort Sampling design

## Abstract

The shape of the length frequency distribution (LFD) is an important input for stock assessments and one of the most important features in studies of fish population dynamics, providing estimates of growth parameters. In practice, oversampling may occur when sampling commercially important species. At times of more and more limited resources, the length sample size can be optimized at some stages of national or regional sampling programmes, without reducing the quality of stock assessments. The main objective of this study is to demonstrate a general distribution-free methodological approach for an optimization of sample size developed as an alternative to both analytical and bootstrap approaches. A novel framework to identify the reduced but still informative sample and to quantify the (dis) similarity between reduced and original samples is proposed. The identification procedure is based on the concept of reference subsample, which represents a theoretical minimal representative subsample that despite smaller sample size still preserves a reasonably precise LFD for certain species. The difference between the original sample and the reference subsample called admissible dissimilarity value (ADV) serves as the upper threshold and can be used to quantify the reliability of derived subsamples. Monte Carlo simulations were conducted to validate the approach under various LFD shapes. We illustrate in case studies how ADV can support to evaluate adequate sampling effort. The case studies focus on length samples from the German commercial vessels fishing for North Sea cod (*Gadus morhua*).

## Introduction

Measurements of body length and weight give direct evidence for growth in fish. The relationship between fish length and weight can be used to convert length to weight and vice versa and is frequently used in stock assessments. Length can be easily and inexpensively measured in the field or laboratory, on live or preserved fish (Busacker et al. 1990), and is therefore a standard parameter in commercial catch sampling. Length sampling is performed in order to evaluate the length distribution of species in catches or landings. Length frequency distributions (LFDs) are an important input for stock assessments ranging from length-based data-limited methods up to full analytical cohort-based assessments, when transformed into age distributions via age-length keys. The LFD can be represented by a single value like mean length (Pennington et al. 2002). Still, the overall shape of LFDs is more important than descriptive statistics like the mean or variance (Gerritsen and McGrath 2007), since it represents the key length patterns for the identification of cohorts and the estimation of growth parameters displayed by species and stocks.

The determination of the appropriate sample size for describing LFD is a frequently discussed problem in fisheries science. The relationships between sample size, intended length intervals, key life-history parameters and number of modes identified in LFDs were investigated by (Erzini 1990). Many studies fitting mixture models to LFDs were conducted (Laslett et al. 2004; Shafii et al. 2010). Singh et al. (2016) have proposed the bootstrap procedure distinguishing the peaks of simulated subsamples and also incorporated an intra-haul correlation in simulation. Gerritsen and McGrath (2007) suggested some rules of thumb for the adequate number of measured individuals required to estimate representative LFDs, based on employing the mean-weighted coefficient of variation under the multinomial distribution assumption. Schultz et al. (2016) and Chih (2010) employed resampling procedures, which indicate the degree of deviation between the original and resampled data based on the mean absolute difference and the total sum of absolute differences in relative frequencies, respectively. The bootstrap simulation procedure for determining an adequate sample size was applied in Gomez-Buckley et al. (1999), which investigated maximum differences between the cumulative distribution function (CDF) of original data and the CDF of random samples of different sizes. Miranda (2007) applied different sample size estimators—histograms, mean length and PSD (proportional stock density)—to describe and quantify distributional length patterns by bootstrapping from original dataset. Commonly, the applied methods include bootstrapping and parametric mixture modelling. However, relying on bootstrap intervals creates a danger of ignoring deviant but still plausible LFDs, as well as accepting practically unsuitable LFDs (e.g. LFD of reduced sample not containing any measurement in some minority length classes from original sample). Moreover, the general technical limitations of bootstrapping methods are their computational expense, as well as complicated way of dependence modelling. On the other hand, the pure statistical/probabilistic approaches alternative to bootstrapping do not guarantee that an obtained reduced sample (i) displays the distributional properties of the original sample well and (ii) includes (if necessary) rare informative observations, i.e. does not artificially simplify the distributional patterns. In particular, the quality of the final mixture model or distribution is highly dependent on the initial parameters and model type selection.

The main objective of this paper is to demonstrate a general distribution-free methodological approach, which was developed as an alternative to both analytical and bootstrap frameworks mentioned above. We propose a novel framework to identify the reduced but still informative sample and to quantify the (dis) similarity between reduced and original samples. The difference from existing approaches is the principle of how the representative reduced samples (or subsamples) are defined, constructed and interpreted. At the core of the approach is the concept of the reference, or benchmark, subsample. Reference subsample in our contexts is the minimal representative subsample that despite smaller sample size still preserves a reasonably precise LFD for certain species. An iterative deterministic subsampling procedure, based on certain conditions, returns a reference subsample, quantifies the difference between the original sample and the reference subsample and provides a threshold value. We have called this threshold an admissible dissimilarity value (ADV). Our approach allows the estimation of the extent of differences between LFDs—or rather empirical CDFs—of the original (target) sample and derived subsamples, by setting the ADV. Of course, the original distribution can also be derived from theoretical models. Preliminary findings were presented at the third Workshop on Optimization of Biological Sampling (WKBIOPTIM3) (see Bitetto et al. (2019)).

The approach might be implemented to support existing length-based approaches in fisheries science and to determine future sampling tasks shared in regional sampling programmes. It may be applied and adapted to many other cases, also outside fisheries science.

Our case study focuses on length samples from the German commercial fleet fishing for North Sea cod (*Gadus morhua*) in the third quarter 2018. We will first describe the case study and dataset used, and then introduce the essential definitions, the formal problem statement and the iterative algorithm description (Materials and methods). The implementation of the algorithm calculated acceptable dissimilarity values, and practical application to reduce sampling effort as well as simulation study is presented and further discussed in Results and discussion.

## Materials and methods

### Data and area description

**Fig. 1:**
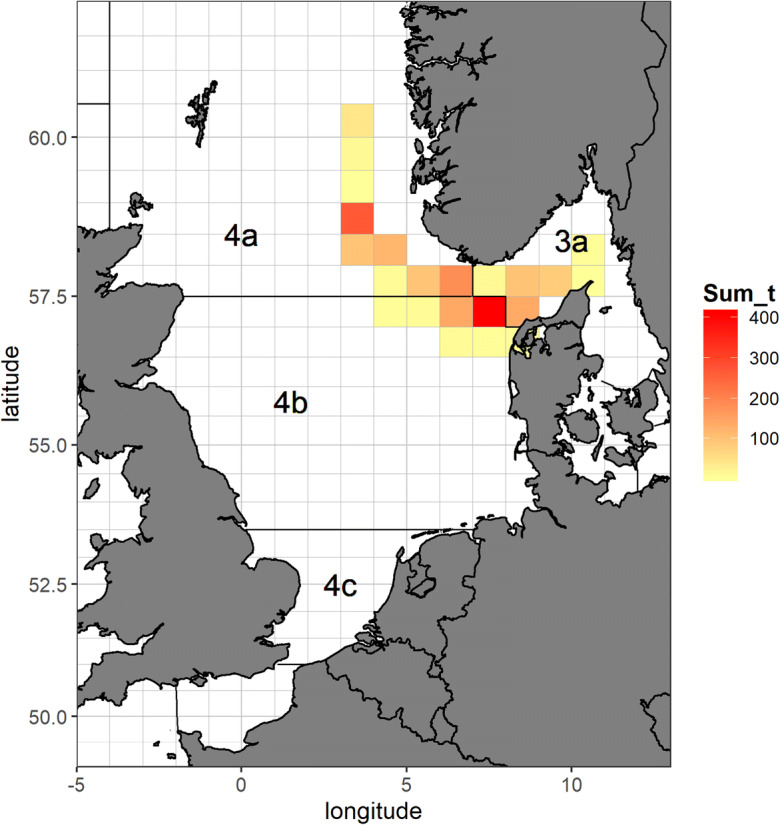
Spatial distribution of German commercial vessels catching cod in the North Sea (sum in metric tonnes per ICES statistical rectangle) for metier OTB_DEF_ > = 120_0_0 combined in the 3rd quarter 2018

**Fig. 2:**
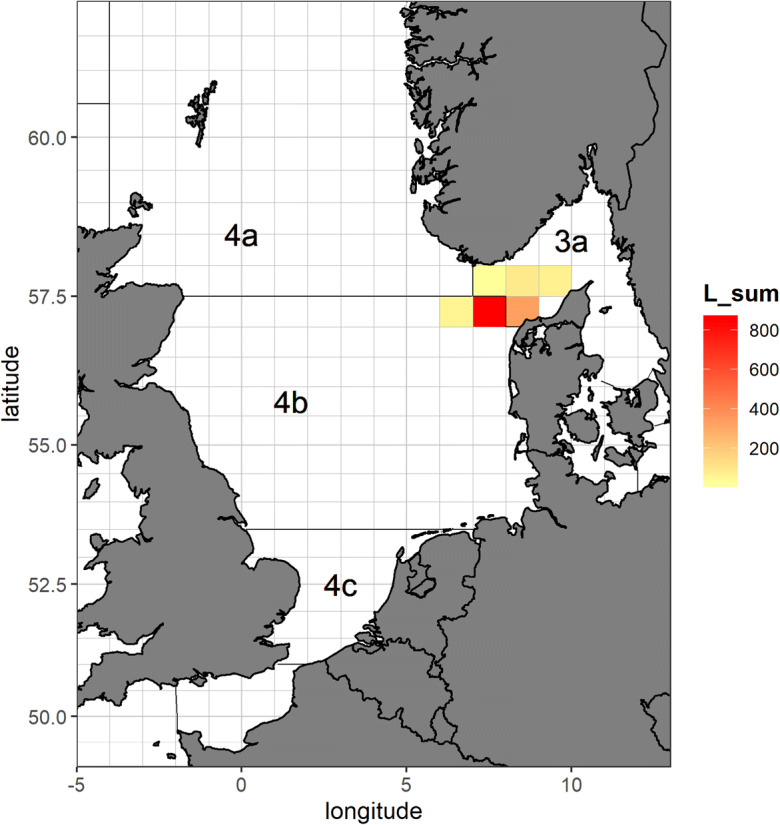
Spatial distribution of unraised length samplings (in absolute numbers of individuals per ICES statistical rectangle) of the German commercial cod fisheries for metier OTB_DEF_ > = 120_0_0 combined in the 3rd quarter 2018

In our study, we focus on German 3rd quarter length frequency data for North Sea cod (*Gadus morhua*) from 2018, in ICES subarea 27.4 (ICES—International Council for the Exploration of the Sea). We selected one métier only for our analysis (OTB_DEF_ > = 120_0_0—otter trawls targeting demersal species with a minimum mesh size 120 mm). This métier operated in the 3rd quarter 2018 in ICES-Divisions 27.3.a, 27.4.a and 27.4.b, targeting cod, saithe and haddock (Fig. 1). Métier is defined as a group of fishing operations targeting a similar assemblage of species, using similar gear, during the same period of the year and/or the same area and which are characterised by a similar exploitation pattern (European decision 2008/949/CE2). Figure 2 demonstrates, consequently, the spatial distribution of cod samples. As basis for the analysis, we extracted the sampled data from the German national database in standard regional database (RDB) format (https://www.ices.dk/marine-data/data-portals/Pages/RDB-FishFrame.aspx) and raised them to the whole catch. This means that measured number of fish at length was expanded to the total number of fish caught in haul, i.e. multiplied by ratios between whole catch weight and the weight of the measured sample. Next to other parameters, the RDB contains biological information sampled by a country during observer trips or at port. In the case of Germany, all data come from observer trips and include wanted (landings) and unwanted (discards and below minimum size landings) catches.

**Fig. 3:**
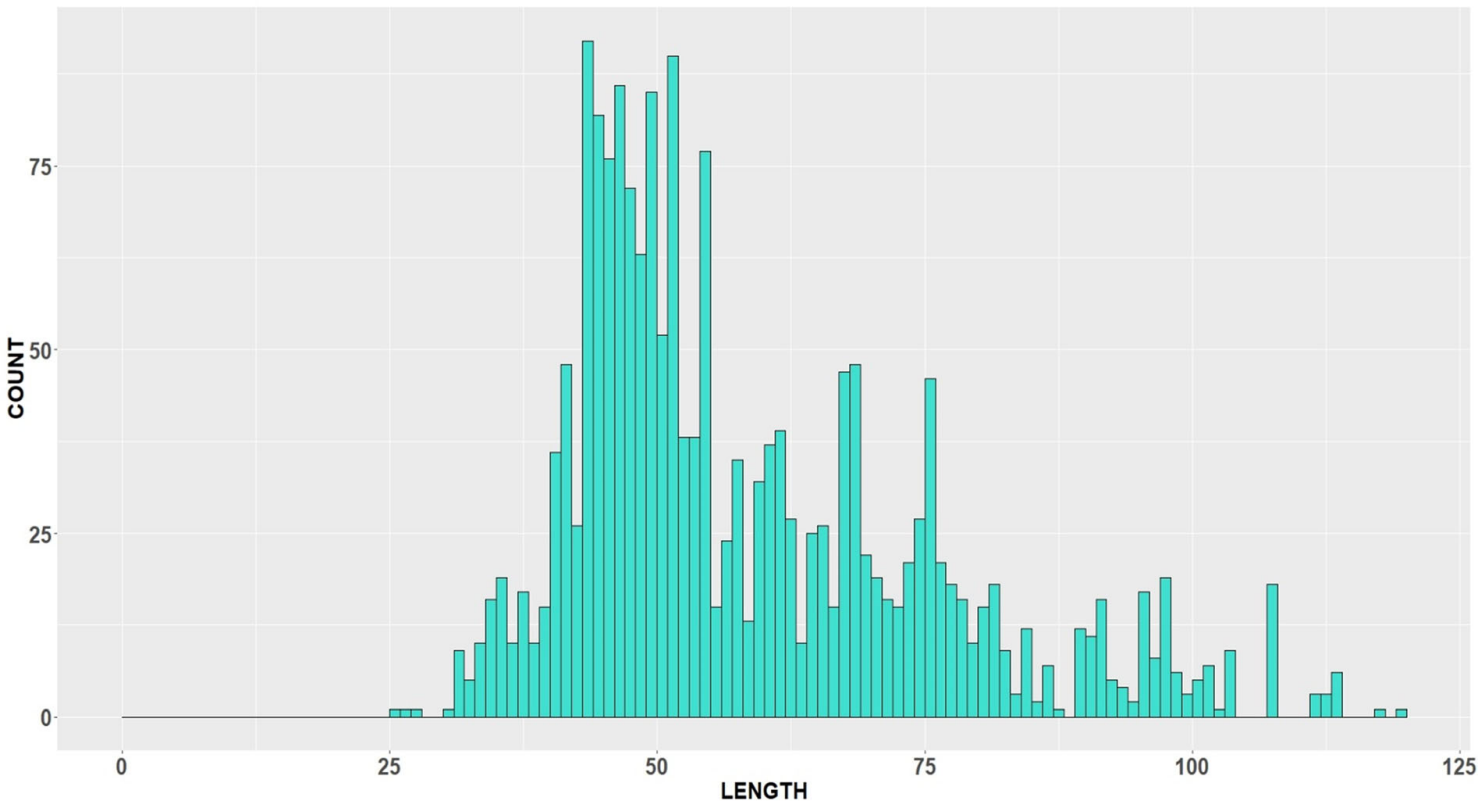
LFD (raised to whole catch) of North Sea cod, obtained by the German commercial observers in the 3rd quarter 2018, in the ICES area 27.4: original sample

A total of 15 observer trips with cod length samples were conducted over the considered period and ICES subareas 27.4 and 27.3.a—Skagerrak (Fig. 2) resulting in approx. 4000 length measurements. However, we would like to avoid combining both ICES areas, since they belong to different strata, and will consider LFD raised to the whole catch in haul and then aggregated by all sampled trips, only from ICES area 27.4 (Fig. 3). Three trips belonging to the same métier OTB_DEF_ > = 120_0_0 were sampled over considered period (Table 1). About 650 individuals were measured over 24 hauls (fishing operations).

**Table 1:** Trips contributing into cod sampling in 3rd quarter 2018 in ICES area 27.4: measured individuals’ unraised and raised to whole catch (in brackets).

Trip code	*n* measured individuals	*n* sampled fishing operations
Trip 1	169 (267)	7
Trip 2	218 (1204)	12
Trip 3	264 (453)	5

### Similarity interpretation

As a starting point, we propose general guidelines as well as a set of terminology to be used when analysing the extent of similarity between LFDs. Generally, LFD is difficult to quantify. Based on the entire original length samples, the aim is to decide on which data subsets from the original sample may be considered as a representative subsample. The arguments and definitions we present below help to find a formal way to verify the dissimilarities between LFDs of original sample and subsample.

The LFD always displays a range of modal length classes or modes (bumps, spikes) and antimodal length classes or antimodes (gaps, dips). Formally, a length class is determined as a mode (antimode) if adjacent length classes exhibit lower (upper) frequency values (i.e. local maxima/minima). For simplicity, it is assumed that if two adjacent length classes could belong to modal (antimodal) classes, i.e. their frequencies are equal and frequencies of preceding and following length classes are higher (lower), we will define the first of them as a modal class. Typically, there is one major mode and few minor (or secondary) modes. It is obvious that in data without sample bias, well-observable modes usually represent the different (strong) year classes of the sampled population, separated by antimodes, which are the boundaries between these age clusters. In other words, the number of modes determines the number of distinct age clusters in length frequencies, and the differences in surrounding modes and antimodes (i.e. amplitudes) indicate rates of detachment of age clusters. Of course, there are also examples where these modes (year classes) and antimodes are very hard (if possible at all) to detect. This is often the case in long-lived (life span of more than 50 years (Cadrin et al. 2010)) and slow-growing fish like redfish of the genus *Sebastes* where the length frequency distributions may be rather “uniform”.

The above-mentioned standard RDB format utilizes data with length rounded to 1 cm (or rounded to 1/2 cm for species like sprat (*Sprattus sprattus*) and herring (*Clupea harengus*) with relatively low maximum length). The standard bandwidth *∆* = 1 cm obviously delivers the maximal number of modes and antimodes present in the dataset and can cause some “spurious” modes and antimodes. To discover which modes/antimodes are robust and to avoid a “spiky” form of the LFD, the original distribution can be smoothed correspondingly by the choice of a bandwidth *∆* > 1.

**Fig. 4:**
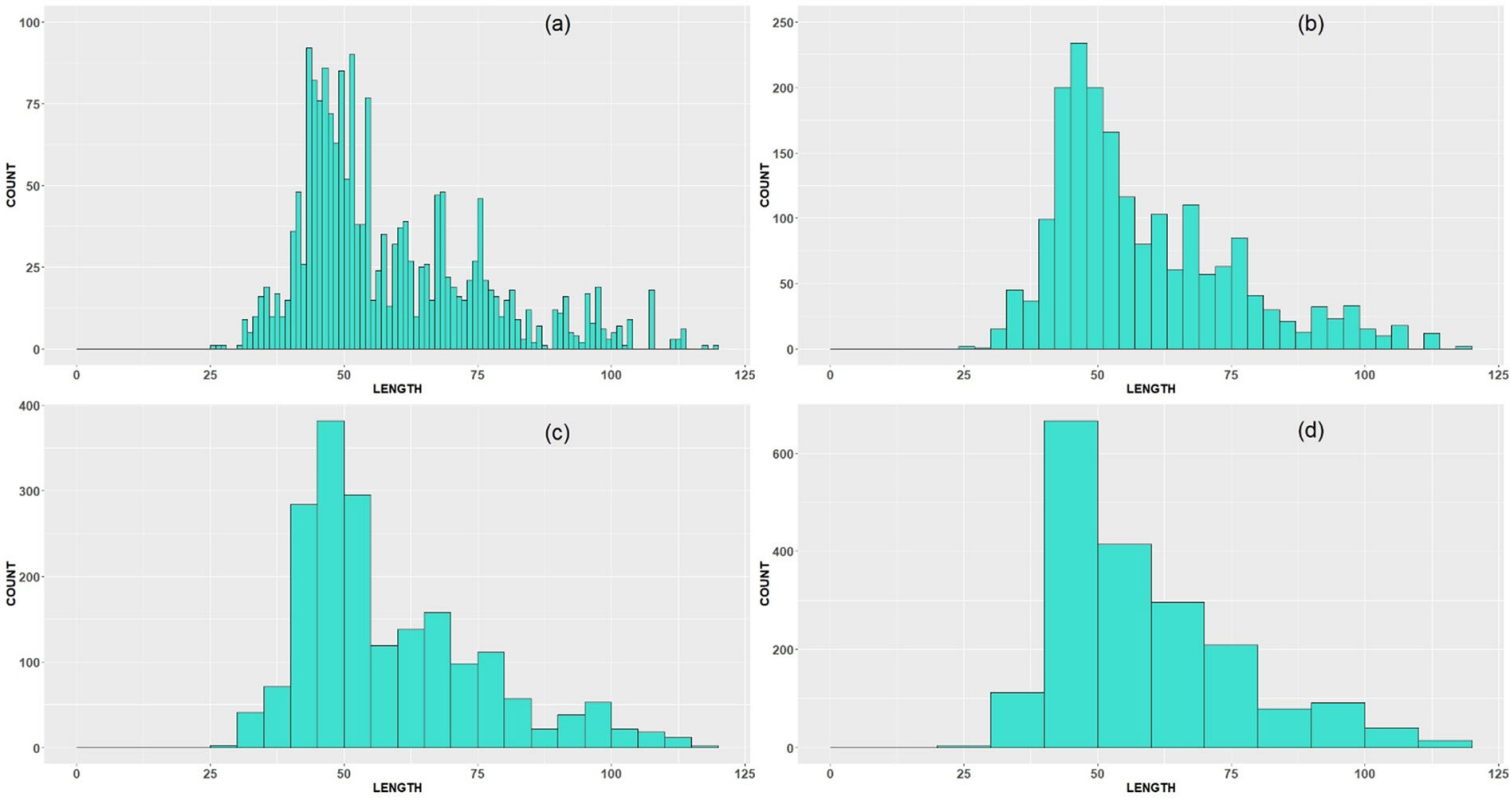
LFD (raised to whole catch) of North Sea cod, obtained by the German commercial observers in the 3rd quarter of 2018, in ICES area 27.4: original sample with different bandwidth and bin origin at 0 **(a)** bandwidth = 1 cm; **(b)** bandwidth = 3 cm; **(c)** bandwidth = 5 cm; and **(d)** bandwidth = 10 cm

Indeed, the shape of length frequency data is completely determined by bandwidth and origin (Scott 1992). To be coherent, we decided always to fix the bin origin at zero. Of course, it can also be chosen as the smallest value in length data or also rounded, respectively. Examples of the 2018 histogram for cod length frequency data with bandwidth of 1 cm suggest more structure in data with many spikes and gaps (Fig. 4). At a bandwidth of 3 cm, the histogram looks smoother, and with a bandwidth equal to 5 cm, it demonstrates just a few modes. The last example (bandwidth of 10 cm) is almost unimodal (excluding a small bump on the right side). It is obvious that bandwidth acts as a smoothing parameter for general patterns in the data. However, during this process, the important detailed features in the data should not get lost. This can be modelled through mixture density (see, e.g. McLachlan and Peel (2000)), but in our case, we stay within a nonparametric framework, instead of relying on model selection, and apply a “biologically reasonable” choice of degree of smoothing *∆*. In Anderson and Newmann (1996), the 1-cm interval was proposed for species reaching maximal 30-cm body length and 2-cm interval for species reaching from 31 to 60 cm. For species with maximal body size 121–150 cm, a 5-cm interval is suggested, and the database provides a maximal cod length equal to 128 cm for the period 2015–2018. Appropriate smoothing helps to make the measuring of dissimilarity between the original sample and the subsample not too sensitive towards changes in subsample shape corresponding to small bumps and gaps in the sample, which could be just incidental for a certain sampling event. The “smoothed” version of the LFDs with a bandwidth of 5 cm is therefore used further in our approach.

Note, that it is not always necessary to be focused on information delivered by all length classes *l* = {*l*_*j*_}, *j* = 1, 2, …*L*. One can choose the most important length classes *l*^*I*^ = {*l*_*j* ∈ *I*_}, *I* ⊆ {1, 2, …, *L*}. Inside these thresholds, the most significant information for a certain application about the LFD shape is provided, while less informative ones are ignored (e.g. very small or very large fish).

The following definition concerns the meaning of significantly distinct as well as robust modes and antimodes in our context.

#### Definition 1.

Let $\overrightarrow{M}={\left({M}_1,{M}_2,\dots \right)}^T$ be modes and $\overrightarrow{A}={\left({A}_1,{A}_2,\dots \right)}^T$ be antimodes of some LFD with bandwidth 1 cm, and ${\overrightarrow{M}}^{smoothed}={\left({M}_1^{\Delta  },{M}_2^{\Delta  },\dots \right)}^T$ be modes and ${\overrightarrow{A}}^{smoothed}={\left({A}_1^{\Delta  },{A}_2^{\Delta  },\dots \right)}^T$ be antimodes of the same LFD with selected bandwidth *∆* > 1 cm, where *∆* = *∆* (max species length).

We define a mode ${M}_i\in \overrightarrow{M}$ as a robust mode on the set of important length classes *l*^*I*^, if

1. $$ {M}_k^{\Delta }\le {M}_i<{M}_k^{\Delta  }+\Delta $$
2. $$ {M}_i=\max \left(M1,M2,\dots \right)\in \left[{M}_k^{\varDelta };{M}_k^{\varDelta }+\varDelta \Big[\right.\left({M}_1,{M}_2,\dots \right) $$
3. *f*(*M*_*i*_) > 0.01 · max(*f*(*M*_1_), *f*(*M*_2_), …),where $$ \overrightarrow{M}\in {l}^I $$.

In the same way, an antimode ${A}_j\in \overrightarrow{A}$ is a robust antimode on the set of important length classes *l*^*I*^, if

1. $$ {A}_r^{\Delta }\le {A}_j<{A}_r^{\Delta  }+\Delta $$
2. $$ {A}_j=\underset{\left({A}_1,{A}_2,\dots \right)\in \left[{A}_r^{\Delta  };{A}_r^{\Delta  }+\Delta  \right[}{\min}\left({A}_1,{A}_2,\dots \right) $$,where $$ \overrightarrow{A}\in {l}^I $$.

According to this, the robust modes and antimodes continue to be present despite length class smoothing and are therefore not suspect to sampling artefacts, “contaminating” the distributional shape. For example, the mode *M*_1_ = 46 cm in Fig. 4 fulfils this criterion, for all bandwidth values *∆* except the last one *∆*=10 cm. Note that *M*_1_ does not deliver the maximal count value for the bandwidth 1 cm (see Fig. 4a) but demonstrates its robustness when the bandwidth increases. In case of the existence of two or more original modes/antimodes within a *∆*-smoothed length class, the dominating one (i.e. maximal/minimal) will be selected as a robust mode/antimode. Moreover, modes having too low frequencies (less than 1% of maximal frequency) are not considered as being robust. However, expert judgment may still be needed to determine the final set of modes and antimodes dependent on the biology of the species and the questions in place.

The next definition provides the formal requirements of statistical-biological similarity between original and subsampled LFDs.

#### Definition 2.

Let $\overrightarrow{M}={\left({M}_1,{M}_2,\dots \right)}^T$ and $\overrightarrow{m}={\left({m}_1,{m}_2,\dots \right)}^T$ be robust modes and $\overrightarrow{A}={\left({A}_1,{A}_2,\dots \right)}^T$ and $\overrightarrow{a}={\left(a,{a}_2,\dots \right)}^T$ be robust antimodes of LFD of the original *S*_*orig*_ = *S*_0_ and reduced *S*_*n*_ samples, respectively, defined on the set of important length classes *l*^*I*^ = {*l*_*j* ∈ *I*_}, *I* ⊆ {1, 2, …, *L*}. We define *S*_0_ and *S*_*n*_ as similar, if:

1. They have the same number of robust modes and antimodes revealed under chosen bandwidth *∆*, i.e. dim($\overrightarrow{m}$) = dim($\overrightarrow{M}$) and dim($\overrightarrow{a}$) = dim($\overrightarrow{A}$).
2. For each corresponding pair *m*_*i*_, *M*_*i*_ and *a*_*j*_, *A*_*j*_:

|*m*_*i*_ − *M*_*i*_|≤ *ε* and |*a*_*j*_ − *A*_*j*_|≤ *ε*, where *ε* = *ε* (max species length).

(3) Amplitudes ratio $\frac{\left|g\left({m}_i\right)-g\left({a}_j\right)\right|}{\left|f\left({M}_i\right)-f\left({A}_j\right)\right|}$ ≥ θ, where *f*(∙), *g*(∙) are the values of the original and reduced sampled LFDs at a point, respectively; *j* ∈ {*i*; *i* + 1}, *i* ∈*ℕ*, 0<θ ≤1.

This definition states that the subsampled dataset has to preserve the structure and specific patterns of the original dataset within *l*^*I*^, revealing the same number of robust modes and antimodes (further also called robust critical points) as well as keeping distinguished differences between adjacent critical values. Locations of critical points for larger specimens do not have to be exact and might vary in some small interval defined by parameter *ε*. Our assumption is that if conditions (1)–(3) are satisfied, two datasets are indistinguishable in both integrated statistical-topological and biological sense.

### Formal problem statement, dissimilarity measure and admissible dissimilarity value (ADV)

Formally, the problem can be defined as follows: determine possible sampling effort reducing scenarios based on subsamples *S* = {*S*_*n*_}, for which a set of conditions/constraints (1)–(3) given in Definition 2, Similarity interpretation, is satisfied. How can a dissimilarity between the original sample and subsample be measured in one number? This should be based on a certain statistical distance.

We propose a new dissimilarity measure, based on the well-known Minkowski metric distance, which includes penalty terms controlling performance of the conditions. The Minkowski metric distance of order *p* is given by:

$$ {L}_p\left(F,G\right)={\left(\sum \limits_{j=1}^I{\left|F\left({l}_j\right)-G\left({l}_j\right)\right|}^p\right)}^{\frac{1}{p}} $$

∙functions (CDFs) of the original and any subsampled datasets, respectively. *F*(*l*_*j*_) and *G*(*l*_*j*_) are their values in length class *l*_*j*_ ∈ *l*^*I*^. It is a very general metric, and using *p* greater than 2 is unusual in practice, since larger values of *p* give greater weights to values in which the CDFs *F* and *G* differ most. In our approach, we employ the well-known Manhattan or 1-Wasserstein distance (Minkowski distance under *p* = 1) or *L*_1_-distance:${L}_1\left(F,G\right)=\sum \limits_{j=1}^I\left|F\left({l}_j\right)-G\left({l}_j\right)\right|$ .

Note that by definition of CDF,

$$ F\left({l}_j\right)=\sum \limits_{k\le j}\frac{f\left({l}_k\right)}{\underset{\_}{f}},G\left({l}_j\right)=\sum \limits_{k\le j}\frac{g\left({l}_k\right)}{\underset{\_}{g}}\bullet \bullet, $$

where *f*(*l*_*j*_), *g*(*l*_*j*_) are the LFD counts at the length class *l*_*j*_ of the original sample and subsample, $\underset{\_}{f}=\sum \limits_{k\in I}f\left({l}_k\right),\underset{\_}{g}=\sum \limits_{k\in I}g\left({l}_k\right)$. The choice of *L*_1_-distance as dissimilarity measure originated from the easy way of visualization (just an area between two empirical CDFs) and lower sensitivity to outliers and abnormal values (e.g. comparing to *L*_2_-distance).

Let $\overrightarrow{V}= sort\left(\overrightarrow{M},\overrightarrow{A}\right)$ and $\overrightarrow{v}= sort\left(\overrightarrow{m},\overrightarrow{a}\right)$ be the sorted increasing sequences of the robust critical points of the original sample and subsample, respectively, and $1\left\{\varPsi \right\}:\varPsi \to \left\{0;1\right\}$ be the indicator function, i.e. $1\left\{\varPsi \right\}=1$ if *Ψ* is true and $1\left\{\varPsi \right\}=0$ otherwise. A dissimilarity between the original sample and subsample is measured by the following distance:

$$ D\left({S}_0,{S}_n\right)={L}_1\left(F,G\right)+{c}_1\cdotp \mathbbm{1}\left\{\dim \left(\overrightarrow{v}\right)\ne \dim \left(\overrightarrow{V}\right)\right\}+{c}_2\cdotp \sum \limits_{i=1}^{d\mathrm{im}\left(\overrightarrow{V}\right)}\max \left(0,\left|{v}_i-{V}_i\right|-\varepsilon\ \right)\cdotp \mathbbm{1}\left\{\dim \left(\overrightarrow{v}\right)=\dim \left(\overrightarrow{V}\right)\right\}+{c}_3\cdotp \sum \limits_{i=2}^{d\mathrm{im}\left(\overrightarrow{V}\right)}\max \left(0,\theta -\frac{\left|g\left({v}_i\right)-g\left({v}_{i-1}\right)\Big)\right|}{\left|f\left({V}_i\right)-f\left({V}_{i-1}\right)\Big)\right|}\right)\cdotp \mathbbm{1}\left\{\dim \left(\overrightarrow{v}\right)=\dim \left(\overrightarrow{V}\right)\right\} $$

where $g\left({v}_i\right)=\underset{\_}{g}\cdotp \left(G\left({v}_i\right)-G\left({v}_{i-1}\right)\right)$, $f\left({V}_i\right)=\underset{\_}{f}\cdotp \left(F\left({V}_i\right)-F\left({V}_{i-1}\right)\right)$, *c*_1_, *c*_2_ and *c*_3_ are some constants. The first term is the *L*_1_-distance between two CDFs as mentioned above, and the three next terms represent penalties, which we impose for violation of constraints (1)–(3), correspondingly. So, if a number of robust critical points in the subsample is different from this number in the original sample (violation of the condition (1)), then $\mathbbm{1}\left\{\dim \left(\overrightarrow{v}\right)\ne \kern0.4em \dim \left(\overrightarrow{V}\right)\right\}=1$ and the distance magnitude equals to *D* = *L*_1_(*F*, *G*) + *c*_1_, so a constant penalty is applied to infeasible LFD of subsample. In the same way, even by the equal number of critical points, the penalty term restrains their shifts: if the shift |*v*_*i*_ − *V*_*i*_| between some *v*_*i*_ and *V*_*i*_ exceeds *ε* (violation of the condition (2)), then max(0, |*v*_*i*_ − *V*_*i*_| − *ε* ) = |*v*_*i*_ − *V*_*i*_| − *ε* and the distance magnitude increase *D* = *L*_1_(*F*, *G*) + *c*_2_ · (|*v*_*i*_ − *V*_*i*_| − *ε* ). The constants *c*_1_, *c*_2_ and *c*_3_ are introduced to define a hierarchy on constraints violation, although they can be put equal to 1. Intuitively, the slightest violation corresponds to the condition (3) and the highest to the condition (1), so that *c*_3_ = 1 and *c*_1_ = 10, for instance. For condition (2), we take *c*_2_ = 2.

Obviously, for *S*_*n*_ ≡ *S*_0_ ,we obtain the lower bound *D* = 0. The upper bound can be provided by a minimally permitted reference subsample representing “the worst case” or rather “minimum sampling effort” case. This is a reference subsample *S*_*ref*_, which still reveals the patterns of the original distributional shape for given parameter values (*∆*, *θ*, *l*^*I*^, *ε*) (i.e. meets conditions (1)–(3)), but cannot be reduced anymore because further subsampling will change the LFD shape. We will call the corresponding distance *D*(*S*_0_, *S*_ref_), where *G*^*ref*^ is the empirical CDF of *S*_ref_, the admissible dissimilarity value (ADV). It represents a threshold (upper limit) to decide on acceptable and unacceptable dissimilarities between LFDs when reducing sampling effort. Thus, all subsamples *S* = {*S*_*n*_} with *D*(*S*_0_, *S*_*n*_) ∈ [0; ADV] can be considered as representative ones in relation to the original target sample, thus, suitable to access the original length distribution information. It is easy to see that ADV = *D*(*S*_0_, *S*_ref_) = *L*_1_(*F*, *G*^*ref*^), since all penalty terms equal to 0.

We should it make clear that the reference subsample is a purely theoretical, unrealistic subsample, which is formally constructed on the base of the conditions (1)–(3) from Definition 2. We use it only to conclude about the reliability of the derived “real-world” subsamples with respect to the original sample.

Next, we describe the iterative subsampling procedure that we apply to obtain reference subsample and compute ADV.

### Reference subsample and iterative subsampling algorithm

In general, conditions (1)–(3) in Definition 2, Similarity interpretation, represent one iteration of the iterative algorithm. Briefly, during the procedure, we remove one length measurement from each length class from *S*_0_, to keep the LFD shape similar to the original one, and check if those conditions are fulfilled. This subsampling process is repeated as long as these conditions are satisfied, otherwise it stops. The resulting subsample represents the reference subsample (as mentioned above, “minimum sampling effort” scenario) delivering a minimally sufficient original distributional information.

Note that the set of parameters (*∆*, *θ*, *l*^*I*^, *ε*) that we apply for construction of the reference subsample can be extended. We introduce the following additional (optional) parameter *γ*, *γ* < *θ*, which indicates a minimally required number per length class in a reference subsample. This can be a fixed number of individuals in each length class (e.g. scalar value *γ* = 10 fishes required for ageing purposes) or relative number (percentage of the original number in each length class, so vector value {*γ*_*j*_}, *j* = 1, 2, …*L*, where *L* is a number of 1-cm length classes). We can say that parameter *γ* reflects the requirements of official national sampling programmes in a certain sense. Without defining of *γ*, some of length classes can be subsampled to zero. Generally, this is not a nuisance—some length classes might be absent in the subsample as well as in the original sample. The role of parameter *γ* is rather managerial than statistical: since the original sample was taken based on certain sampling regulations (like minimal fish number per length class), and a constructed subsample is its “substitution”, it should adopt all characteristics of the original sample. Of course, the parameter *γ* can be also set to zero.

Formally, the iterative algorithm scheme can be described as follows:

1. Use the standard RDB data with length rounded to 1 cm as basic input data.
2. Select bandwidth *∆* and important length classes *l*^*I*^ if desired; identify corresponding robust critical points in the original sample under *∆* on the set *l*^*I*^.
3. Set remaining parameters {*θ*, *γ*, *ε*} .
4. Remove one length measurement from each length class in *l*^*I*^, and see whether conditions (1)–(3) are satisfied. If yes, repeat the step. If no, go back to the previous subsample and stop. If a number of length measurements in some length class reaches value *γ*, subsampling of this length class stops either, but subsampling of other length classes proceeds further until the conditions are met.

According to the above described procedure, under *γ* = 0 at each iteration step, subsample size equals *n* − *i* · *I*, where *n* is original sample size, *i* is the iteration, and *I* is a number of important length classes. Mathematically speaking, the concept of reference subsample can be defined as a limit of a sequence of these subsamples—a bound that cannot be crossed. Each preserves a general modal/antimodal structure revealed in original sample. Note that subsamples are constructed deterministically, i.e. without random sampling, since random selection does not guarantee a desired result. Indeed, a random elimination can deliver a subsample, which has a potential of being further reduced, but cannot, because it already violates conditions (1)–(3). An extreme example would be a subsample, where only a few length classes are substantially reduced, but the rest remains untouched. Then, adopting such a subsample as a reference would be confusing. The subsampling bootstrap, generating multiple subsamples, might be implemented here but is too computationally prohibitive to be practical and can induce some distributional uncertainty. Still, employing a specific type of a random sampling in the algorithm is a challenging task that we are currently working on. The present study involves only a deterministic type of subsampling procedure.

The corresponding generic iterative algorithm was implemented in the R-5.3.1 software tool.

## Results and discussion

### Reference subsample construction and corresponding ADVs

To illustrate the application of the above-described iterative algorithm in a case study, the required set of input parameters {*∆*, *θ*, *l*^*I*^, *γ*, *ε*} need to be chosen:

- *∆* = 5, for all examples below. The parameter *∆* is needed to decide if a certain critical point is robust or no but doesn’t really related to any termination rule in the further subsampling process. Therefore, the histograms we present below refer only to 1 cm bin width, not 5 cm.
- *γ*=0.2 ${n}_{l_j}$, where ${n}_{l_j}$ is a number of individuals in the length class *l*_*j*_ of original sample (in other words, any length class can be further subsampled, until this contains more than 20% of length measurements from original sample). Note that the length classes with low number of length measurements (${n}_{l_j}<5$) will be then deleted by reference subsample construction. Thus, if desired a constraint $$ \gamma =\left\{\begin{array}{c}0.2\ {n}_{l_j}\kern0.5em ,\mathrm{for}\ {n}_{l_j}\ 5,\\ {}{n}_{l_j},\kern0.5em \mathrm{for}\ {n}_{l_j}<5,\kern0.5em \end{array}\right. $$ can be set. We use this constraint for all variants below.
- For important length classes *l*^*I*^ and parameters *ε* and *θ*, we consider in our example three variants:
- Variant (a):
  - *l*^*I*^: all length classes presented in the original sample
  - *ε* = 0
  - *θ* = 0.9 (the amplitude differences in reference subsample should be kept at least at the level of 90% of original ones)
- Variant (b)
  - Length classes, which don’t contain large (> 90 cm) specimens, *l*^*I*^ = ]0; 90]
  - *ε* = 1
  - *θ* = 0.9
- Variant (c):
  - only moderate length classes, *l*^*I*^ = [40; 80]
  - *ε* = 3
  - *θ* =0.7

**Fig. 5:**
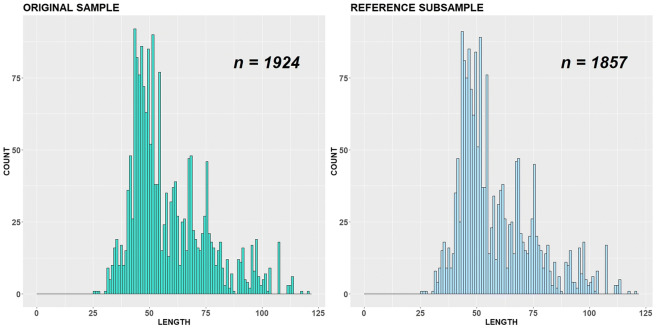
Original sample vs reference subsample with bandwidth = 1 cm, variant (a)

**Fig. 6:**
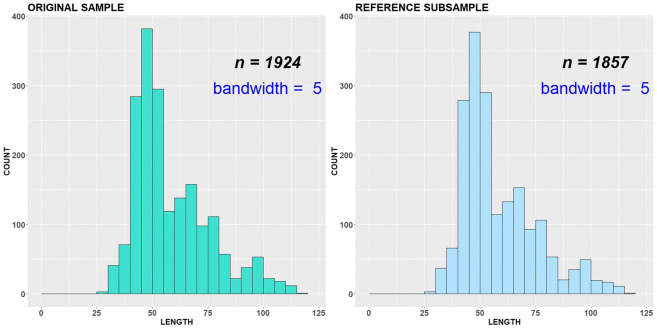
Original sample vs reference subsample with bandwidth = 5 cm, variant (a)

The original dataset includes the length measurements for year 2018 (Fig. 5). The robust modes/antimodes determined for all length classes under smoothing parameter *∆* = 5 are equal to 46, 68, 75, 97 and 58, 72, 85, respectively (Figs. 5 and 6).

**Fig. 7:**
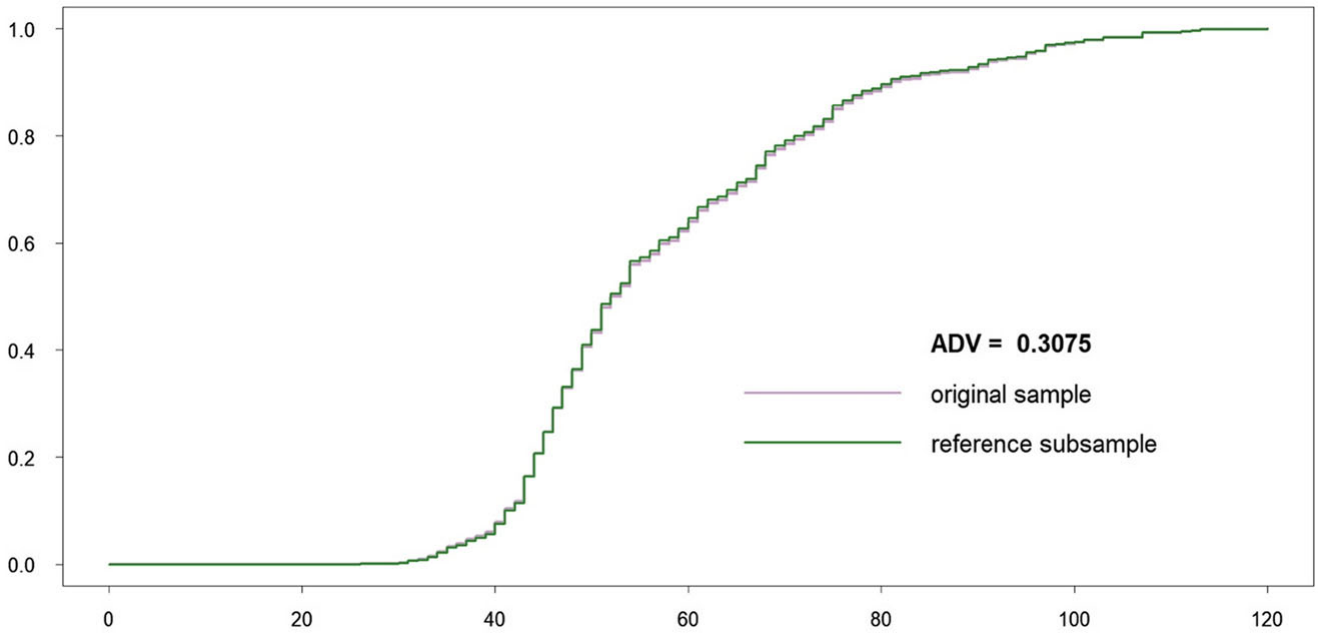
Original sample vs reference subsample: empirical CDFs and ADV corresponding to the variant (a)

All length classes are considered as important and involved in the ADV computation. Both original and subsampled datasets are almost indistinguishable visually, because the reference subsample includes only 67 individuals less than the original sample. The procedure stopped because a number of robust critical points were changed during subsampling. The length class 102 cm became new robust antimode. The corresponding empirical CDFs (*F* and *G*^ref^) as well as ADV are displayed in Fig. 7.

**Fig. 8:**
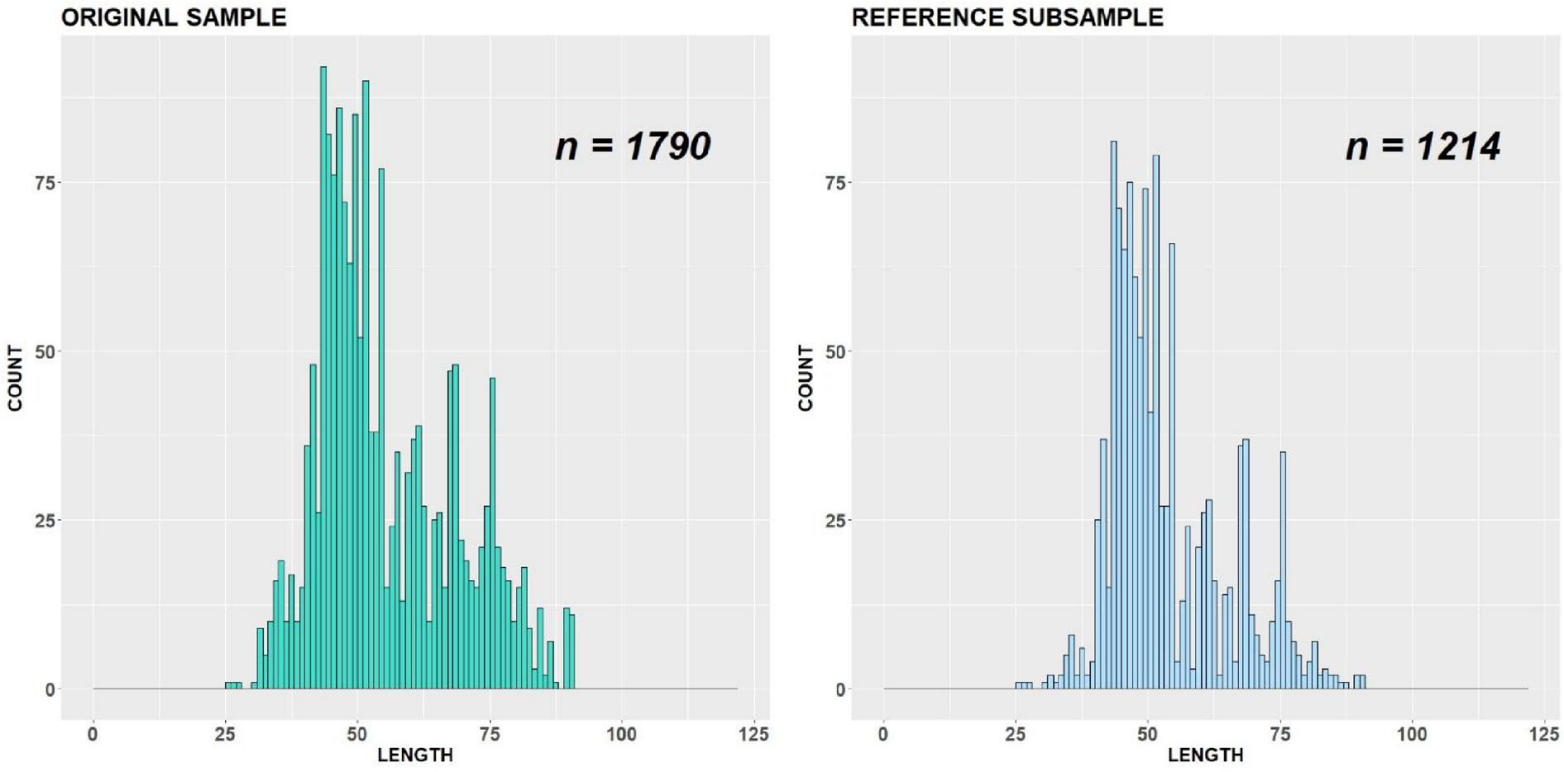
Original sample vs reference subsample with bandwidth = 1 cm, variant (b)

**Fig. 9:**
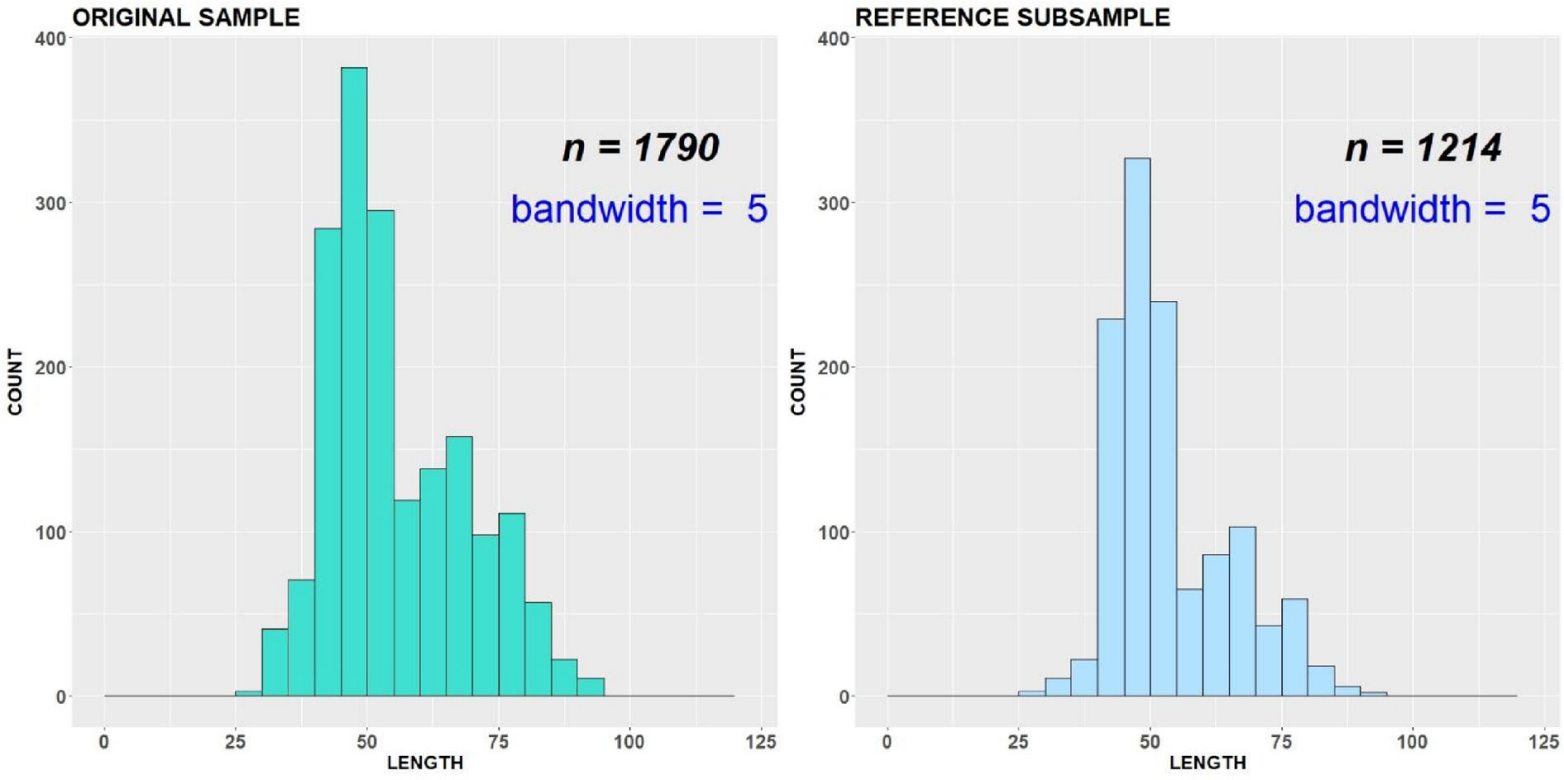
Original sample vs reference subsample with bandwidth = 5 cm, variant (b)

**Fig. 10:**
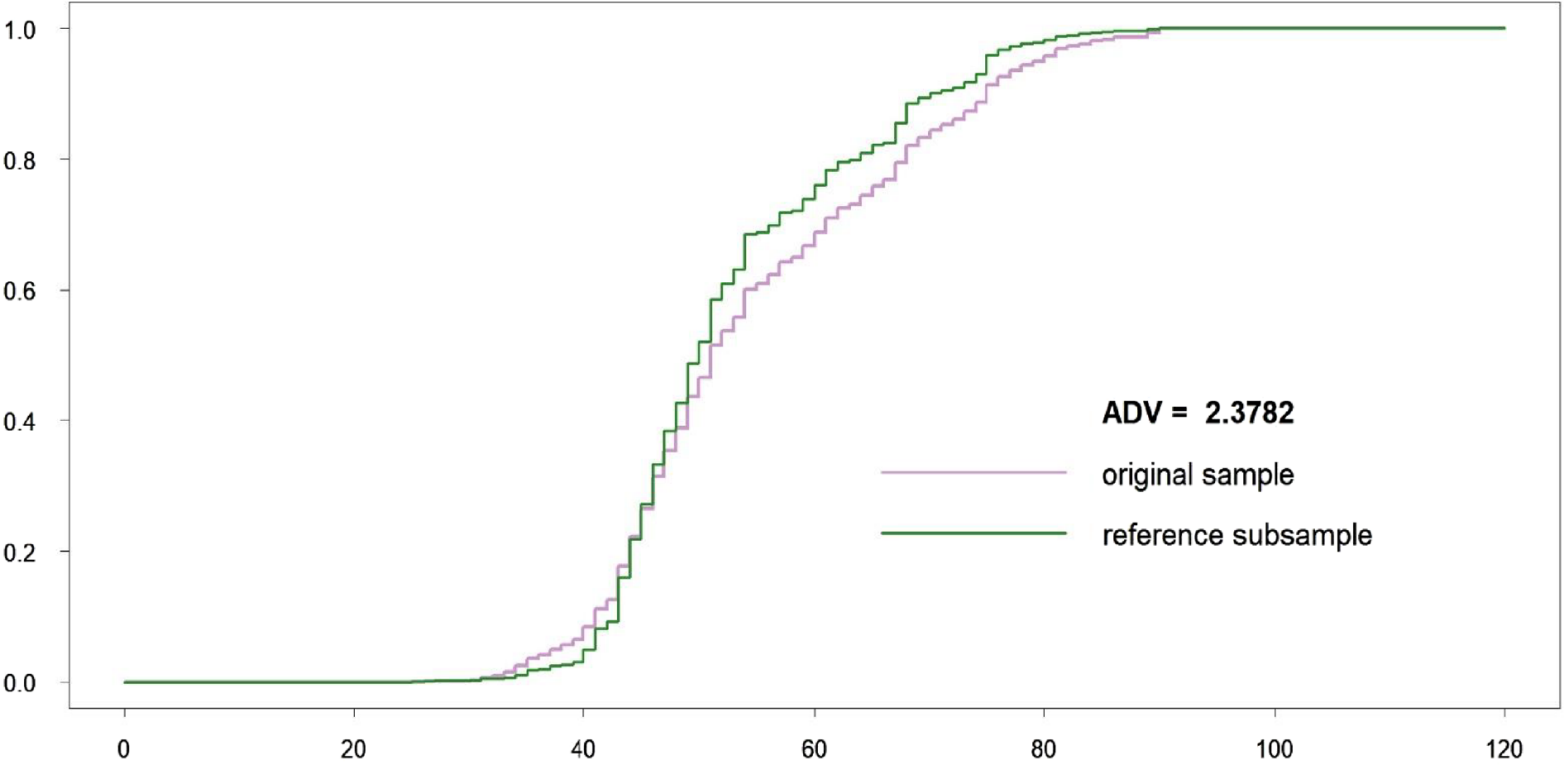
Original sample vs reference subsample: empirical CDFs and ADV corresponding to the variant (b)

For variant (b), we cut off our original sample at length class 90 cm (Figs. 8, 9 and 10). Obviously, it covers a smaller number of critical points than in variant (a) and consequently has a smaller number of restrictions. Now it is allowed to remove 576 measured individuals from important length classes of the original sample without changing distributional patterns. Note that in this case, the reason of iterations stop was different from variant (a). One of the critical points, namely antimodal length class 58 cm, shifted to a new antimode at class 55 cm. This happened because the number of measurements at length class 58 cm in the subsample reached a minimally permitted number of individuals fixed by parameter *γ* and cannot be reduced further. So, at some iteration step, the count numbers at length classes 55 cm and 58 cm became equal, and the value 55 cm as first in order for the *∆*-interval [55; 59] is nominated as a new antimode instead of 58 cm. As we put *ε* = 1 < 58 − 55 = 3 for the variant (b), this has defined the stopping rule here.

**Fig. 11:**
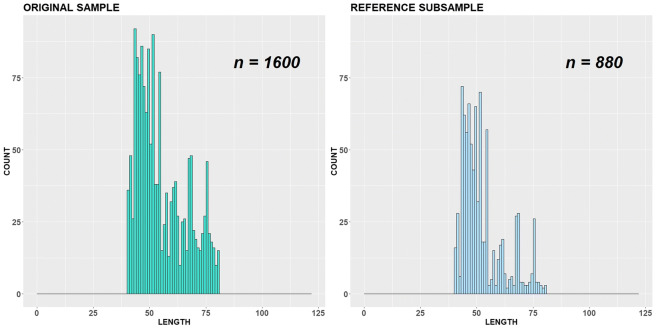
Original sample vs reference subsample with bandwidth = 1 cm, variant (c)

**Fig. 12:**
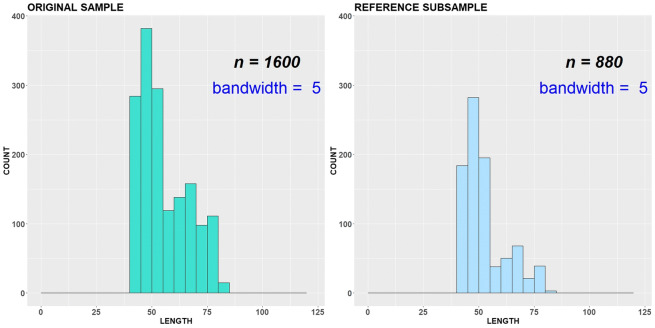
Original sample vs reference subsample with bandwidth = 5 cm, variant (c)

**Fig. 13:**
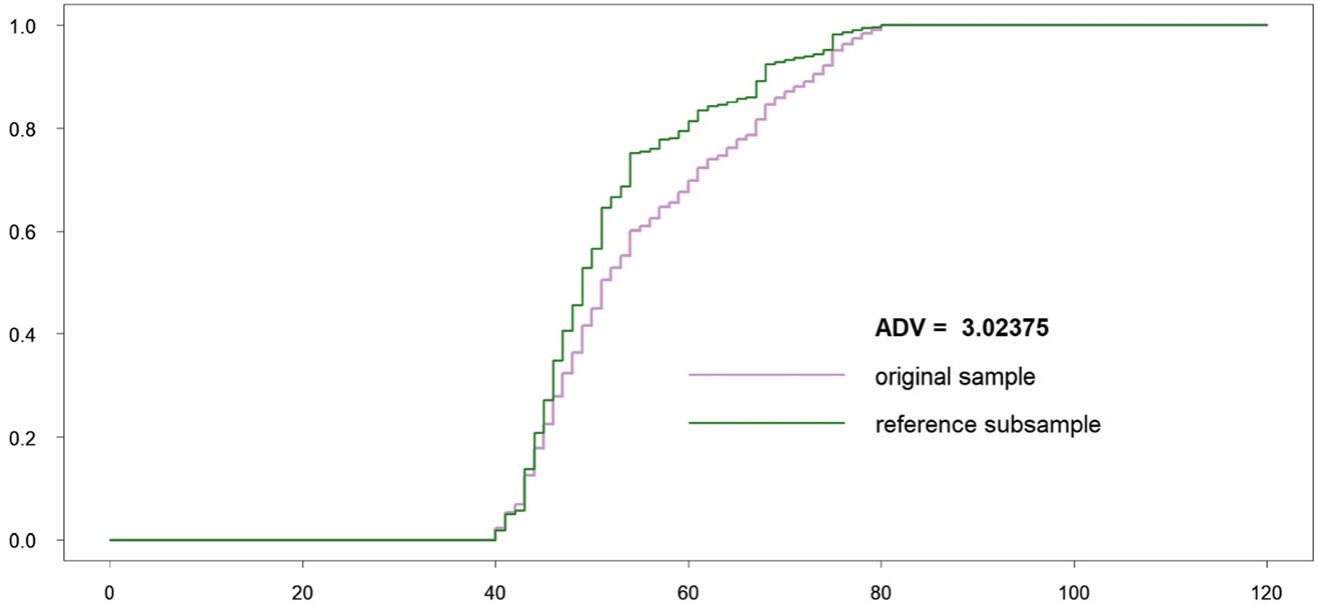
Original sample vs reference subsample: empirical CDFs and ADV corresponding to the variant (c)

Finally, in variant (c), we considered only the length classes from 40 to 80 cm (Figs. 11, 12 and 13). The difference here is that the critical points are not kept constant during the subsampling and can “shift” inside the interval defined by parameter *ε* = 3, that is, 3 cm to both sides. Therefore, even when the antimodal length class 58 cm will be changed to 55 cm during the subsampling, this does not stop the procedure. The stopping in this case was caused by the amplitude ratio condition: the amplitude ratio between the second robust mode and the first robust antimode reached a value less than defined by *θ* = 0.7. The number of removed individuals from important length classes is equal to 720.

### Reducing sampling effort: various scenarios of sampling units exclusion

Obtained in the previous subsection, the ADVs allow us to identify representative subsamples, which we construct by sequential hierarchical elimination of sampling units at different levels—trips, hauls and measured individuals—from the real cod length sample *S*_0_ given in Table 1. However, for the most at-sea sampling situations, effort reducing in practice can be achieved rather by omitting either hauls or individuals, not the entire trips. Still, the example illustrates various possible alternatives of sampling effort reductions.

Three trips occur in the third quarter of year 2018 participating in North Sea cod sampling in area 27.4 (Table 1). Table 2 shows the elimination results for the case of reference subsamples defined by variant (c) in Reference subsample construction and corresponding ADVs. As accompanying magnitudes, the mean length and its standard error were computed from a simple random effects model (Helle and Pennington 2004) and is given by:

$$ {s}_{i,h,t}=\mu +{\varepsilon}_t+{\varepsilon}_h+{\varepsilon}_r, $$

where *s*_*i*, *h*, *t*_ is the length of fish *i* in trip *t* and haul *h*, *μ* is the population mean length of all fishes in the catch, and *ε*_*t*_, *ε*_*h*_ and *ε*_*r*_ are random components describing trip, haul and within-haul variations, respectively. Consequently, $\mathrm{Var}\left[{s}_{i,h,t}\right]={\sigma}_t^2+{\sigma}_h^2+{\sigma}_r^2$, if the independence of *ε*_*t*_, *ε*_*h*_ and *ε*_*r*_ is assumed and their variances equal ${\sigma}_t^2$, ${\sigma}_h^2$ and ${\sigma}_r^2$, respectively.

**Table 2:** Sampling units elimination results, for reference subsample variant (c)

*S*_*n*_	Eliminated sampling units	Distance *D*	Sample size, all length classes/length classes *l*^*I*^ = [40; 80]	Mean length/standard error of mean length in length classes *l*^*I*^ = [40; 80]
*S*_0_	None	0	1924 / 1600	55.99 / 1.77
Trips elimination				
*S*_1_	Trip 1	0.4744	1657/1428	54.14/1.08
*S*_2_	Trip 2	12.8497	720/525	57.65/1.92
*S*_3_	Trip 3	0.6616	1471/1247	56.31/2.87
*S*_4_	Trip 1 + Trip 3	11.3917	1204/1075	53.63/1.54
Hauls elimination				
*S*_1.1_	• Trip 1 • Hauls less than 3 tonnes total catch weight from remained trips	4.9655	695/612	54.00/1.38
*S*_1.2_	• Trip 1 • Hauls less than 1.5 tonnes total catch weight from remained trips	10.7946	1268/1071	54.77/1.09
*S*_1.3_	• Trip 1 • Night time hauls (21:00 ÷ 03:00) from remained trips	0.3176	1573/1352	54.85/1.36
*S*_3.1_	• Trip 3 • Hauls less than 3 tonnes total catch weight from remained trips	10.92	822/681	56.55/2.82
*S*_3.2_	• Trip 3 • Hauls less than 1.5 tonnes total catch weight from remained trips	10.8254	1082/890	56.73/2.47
*S*_3.3_	• Trip 3 • Night time hauls (21:00 ÷ 03:00) from remained trips	0.4819	1390/1196	56.93/3.16
Individuals elimination				
*S*_1.3.1_	• Trip 1 • Night time hauls (21:00 ÷ 03:00) from remained trips • 50% of individuals from remained hauls	5.0786 (median)	788/677.75 (mean)	54.90/1.48 (mean)
*S*_1.3.2_	• Trip 1 • Night time hauls (21:00 ÷ 03:00) from remained trips • 20% of individuals from remained hauls	4.2839 (median)	1259/1082.15 (mean)	54.86/1.39 (mean)
*S*_1.3.3_	• Trip 1 • Night time hauls (21:00 ÷ 03:00) from remained trips • 10% of individuals from remained hauls	0.3680 (median)	1417/1218.25 (mean)	54.85/1.36 (mean)

Removing either Trip 1 or Trip 3 (subsamples *S*_1_ or *S*_3_; Table 2) does not affect the distributional shape, and we still stay below the ADV with distances *D* equal to 0.47 and 0.66, respectively. So, conditions (1)–(3) are satisfied, and penalty terms in distance expression vanished. However, eliminating Trip 2 (subsample *S*_2_) changes the distribution drastically and is therefore unacceptable. Deleting both Trip 1 and Trip 3 (subsample *S*_4_) removes the last robust modal length class 75 cm, rejecting the existence of a pronounced larger length group that appears in the original sample (violation of the condition (1)). This makes the original and subsampled distributions dissimilar and diminish the information delivered by the considered subsample. The lowest bias compared to *S*_0_ was produced by eliminating Trip 3 (subsample *S*_3_), but the subsample *S*_1_ gives us the lowest variance.

Next, we will continue the sampling intensity reduction and try to analyse the effect of the elimination of fishing operations (hauls) within trips. We consider now the length datasets provided by Trip 2 and Trip 3 (subsample *S*_1_) as well as by Trip 2 and Trip 1 (subsample *S*_3_) and assume that observer deployment is not needed for fishing operations where total catch weight is lower than 3 tonnes. Of course, this selection criterion cannot guarantee sufficient quality of samples, because the information on within-haul distribution is unknown. We apply it to illustrate a possibility of managing (reducing) observer’s workload stress. Seven fishing operations conducted during both Trip 2 and Trip 3 and containing cod match this assumption, and their removal changes LFD (Table 2, subsample *S*_1.1_), as *D*(*S*_0_, *S*_1.1_) > > ADV = 3.02. Namely, one of antimodes shifts from the value 58 cm to the value 63 cm (violation of the condition (2)), and some of the amplitudes ratios go below the *θ* = 0.7 (violation of the condition (3)).

Lowering a boundary for the total catch weight per fishing operation (now 1.5 tonnes instead of 3 tonnes, see subsample *S*_1.2_, 2 fishing operations are removed) reveals that even a low standard error estimate for mean length does not guarantee that the LFD shape is preserved: the largest robust mode (75 cm) disappears, so the severely penalized violation of condition (1) kicks the distance value *D* out of the interval [0; ADV]. The probable reason is that 2 removed hauls with lower catch weight do not include larger length classes. The subsamples *S*_3.1_ and *S*_3.2_, where 6 and 2 hauls were removed, respectively, show this unsuitable result as well.

Reducing sampling effort to the daytime (from 03:00 to 21:00) from subsamples *S*_1_ and *S*_3_ results in the elimination of 2 sampled fishing operations from *S*_1_ (one from Trip 2 and one from Trip 3), as well as from *S*_3_ (also one within each trip). Obtained subsamples *S*_1.3_ and *S*_3.3_ (Table 2) display LFD patterns similar with *S*_0_. But the mean length estimation appears to be more reasonable for subsample *S*_1.3_, and it is used for further consideration.

Finally, the dataset *S*_1.3_ can be used for further reducing the number of individuals required to be measured by observers. A resampling analysis to investigate the effect of such reductions was proposed in (Wang et al. 2019). Following a similar resampling procedure, we draw randomly from each haul 50%, 80% and 90% of measured individuals (subsamples *S*_1.3.1_, *S*_1.3.2_ and *S*_1.3.3_ of Table 2, respectively). Since we have to rely on simulation results here, we report the resulted median values of distance *D* for the entire *k* = 3000 replications (mean values can be more affected by extreme values of *D*, obtained in some replications). Subsample *S*_1.3.3_ demonstrates the best result. Bold frames in the Table 2 select subsamples obtained by sequential elimination of sampling units.

Just for the sake of comparison, we perform a two-sample Kolmogorov-Smirnov test of the obtained reference subsamples for variants (a)–(c), Reference subsample construction and corresponding ADVs, versus the sample containing length measurements present in the original sample but absent in the reference subsample, i.e. compliment of the reference subsample. This is similar with a test comparing distributions of the original sample and reference subsample. Really, the reference subsample is a part of the original sample, and if there is difference between them, this can be caused only by the compliment of the reference subsample with respect to the entire original sample. The test resulted in small *p* values indicating rejection of the null hypothesis that subsample distributions are identical with the original sample for variants (b) and (c). The *p* value for variant (a) equals to 0.007; thus, the null hypothesis can be accepted only at the level 0.001. This result was expected, since the Kolmogorov-Smirnov test tends to emphasize the region near the middle peaks of distribution—the length classes where most of the length measurements were removed. This demonstrates a difference to our approach, where the dissimilarity between the original sample and reference subsample are based on biological and statistical-topological components covered by parameters {*∆*, *θ*, *l*^*I*^, *γ*, *ε*}. We have to note, however, that in general, the Kolmogorov-Smirnov test is not very reliable on binned data, if the bins are not small enough. However, most of the length measurements of fish are conducted by 1-cm length class intervals, so the unbinned (continuous) data are not available. Besides this, many standard statistical tests assume a random sample of individual fishes and, hence, cannot be really applied for comparison of LFDs.

### Simulation study

**Fig. 14:**
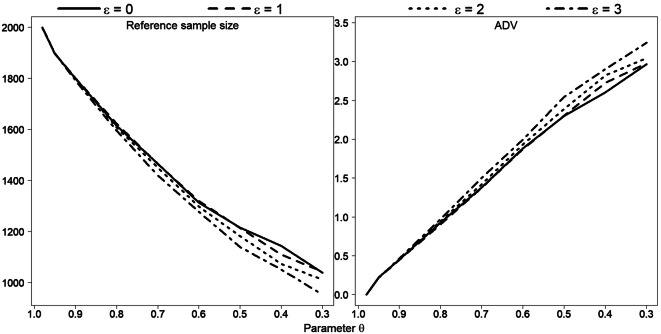
Reference subsample size and ADV for various combinations of parameters *ε* and *θ*. Each point represents the average of 1000 simulation runs of data ${\boldsymbol{y}}_{\boldsymbol{i}}\sim \frac{\mathbf{1}}{\mathbf{4}}\ \boldsymbol{N}\left(\mathbf{40},\mathbf{5}\right)+\frac{\mathbf{1}}{\mathbf{2}}\ \boldsymbol{N}\left(\mathbf{70},\mathbf{5}\right)+\frac{\mathbf{1}}{\mathbf{4}}\ \boldsymbol{N}\left(\mathbf{100},\mathbf{5}\right)$**,** ***i*** **= 1,** …**,2000**

**Fig. 15:**
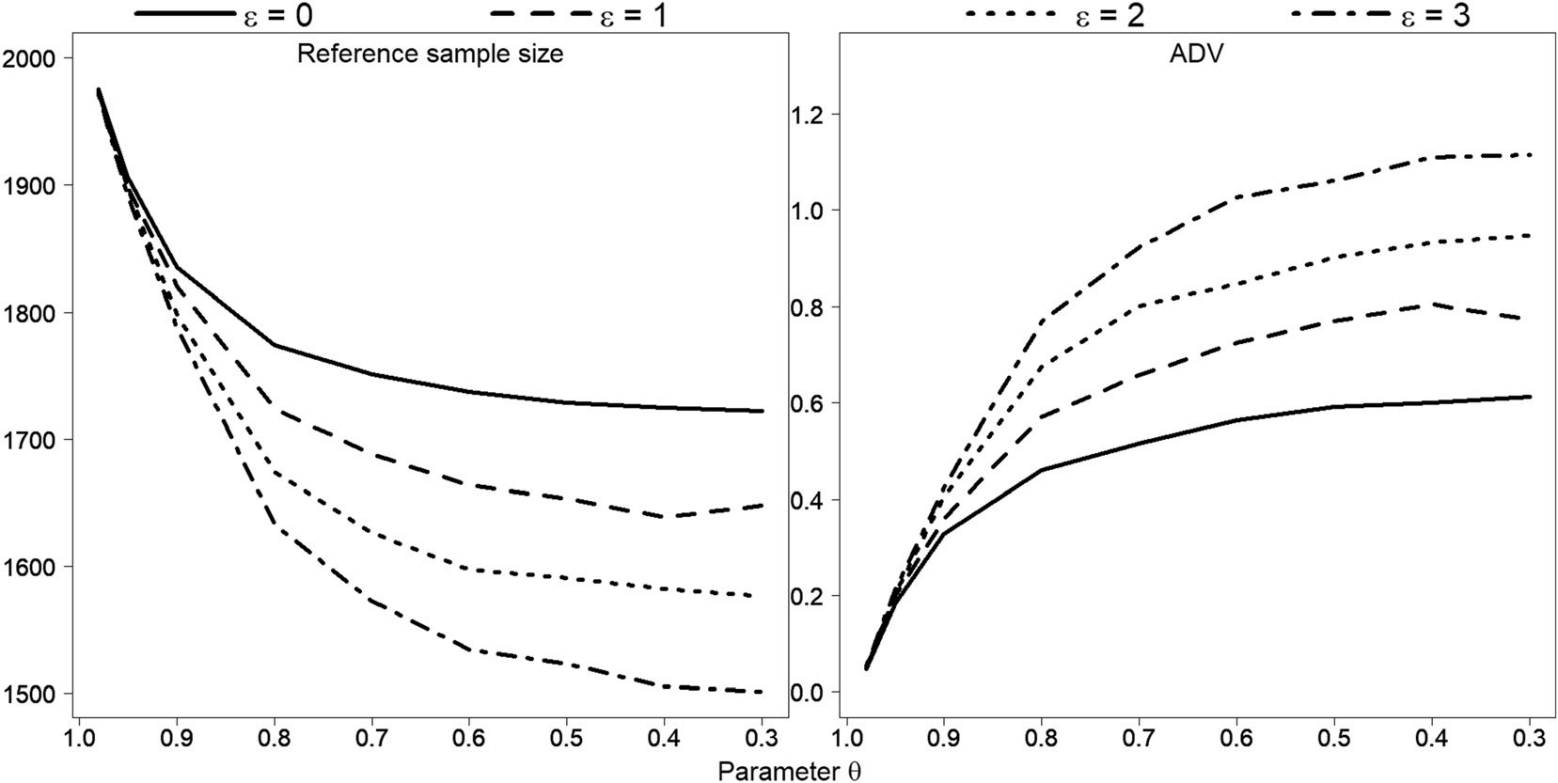
Reference subsample size and ADV for various combinations of parameters *ε* and *θ*. Each point represents the average of 1000 simulation runs of data ${\boldsymbol{y}}_{\boldsymbol{i}}\sim \frac{\mathbf{1}}{\mathbf{4}}\ \boldsymbol{N}\left(\mathbf{40},\mathbf{7}\right)+\frac{\mathbf{1}}{\mathbf{2}}\ \boldsymbol{N}\left(\mathbf{70},\mathbf{7}\right)+\frac{\mathbf{1}}{\mathbf{4}}\ \boldsymbol{N}\left(\mathbf{100},\mathbf{7}\right)$**,** ***i*** **= 1,** …**,2000**

**Fig. 16:**
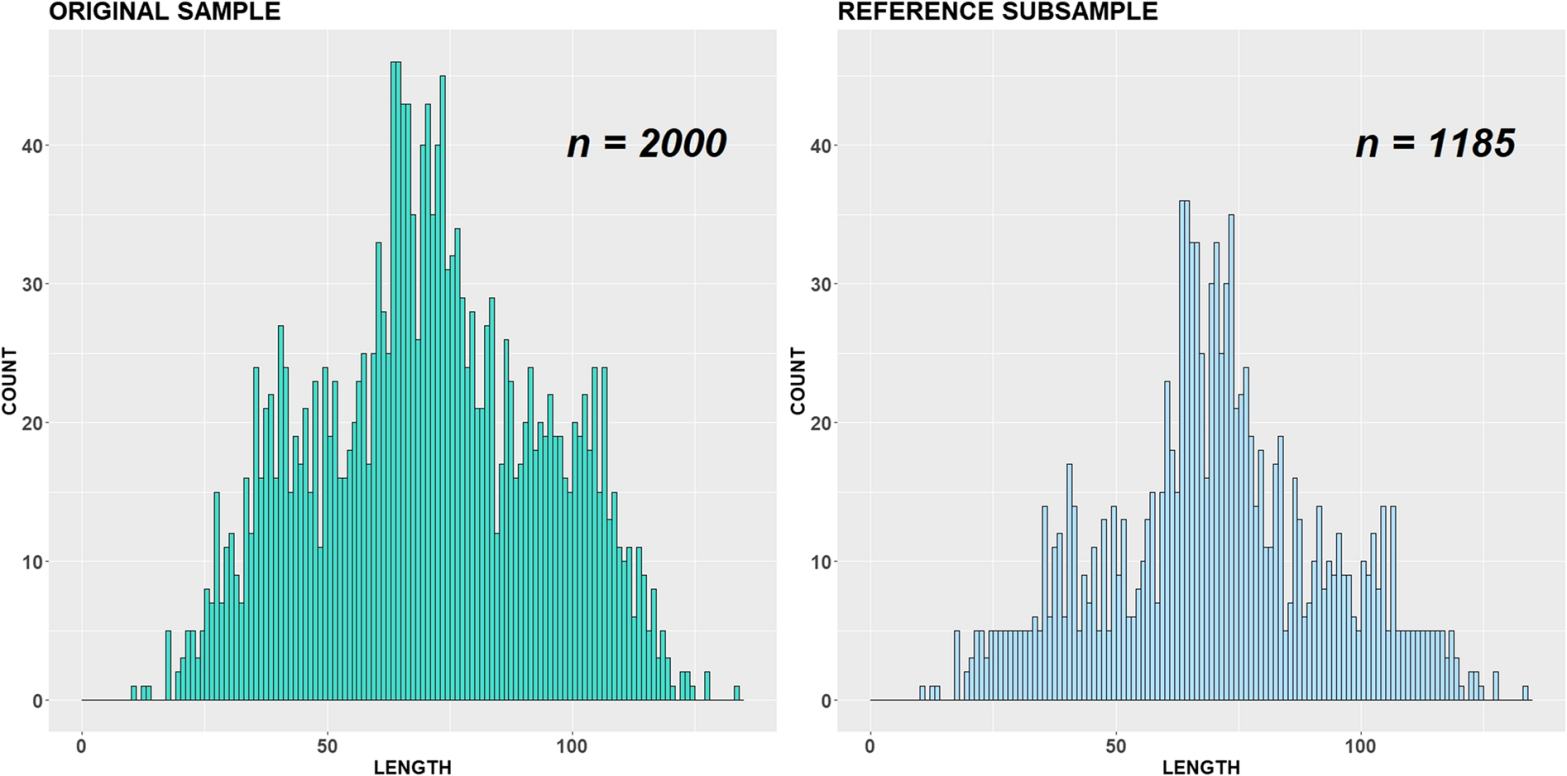
Original sample y and reference subsample under *θ* = 0.95 and ***ε*** **= 0:** $\mathbf{y}\sim \frac{\mathbf{1}}{\mathbf{4}}\ \boldsymbol{N}\left(\mathbf{40},\mathbf{10}\right)+\frac{\mathbf{1}}{\mathbf{2}}\ \boldsymbol{N}\left(\mathbf{70},\mathbf{10}\right)+\frac{\mathbf{1}}{\mathbf{4}}\ \boldsymbol{N}\left(\mathbf{100},\mathbf{10}\right)$**,** ***i*** **= 1,** …**,2000**

**Fig. 17:**
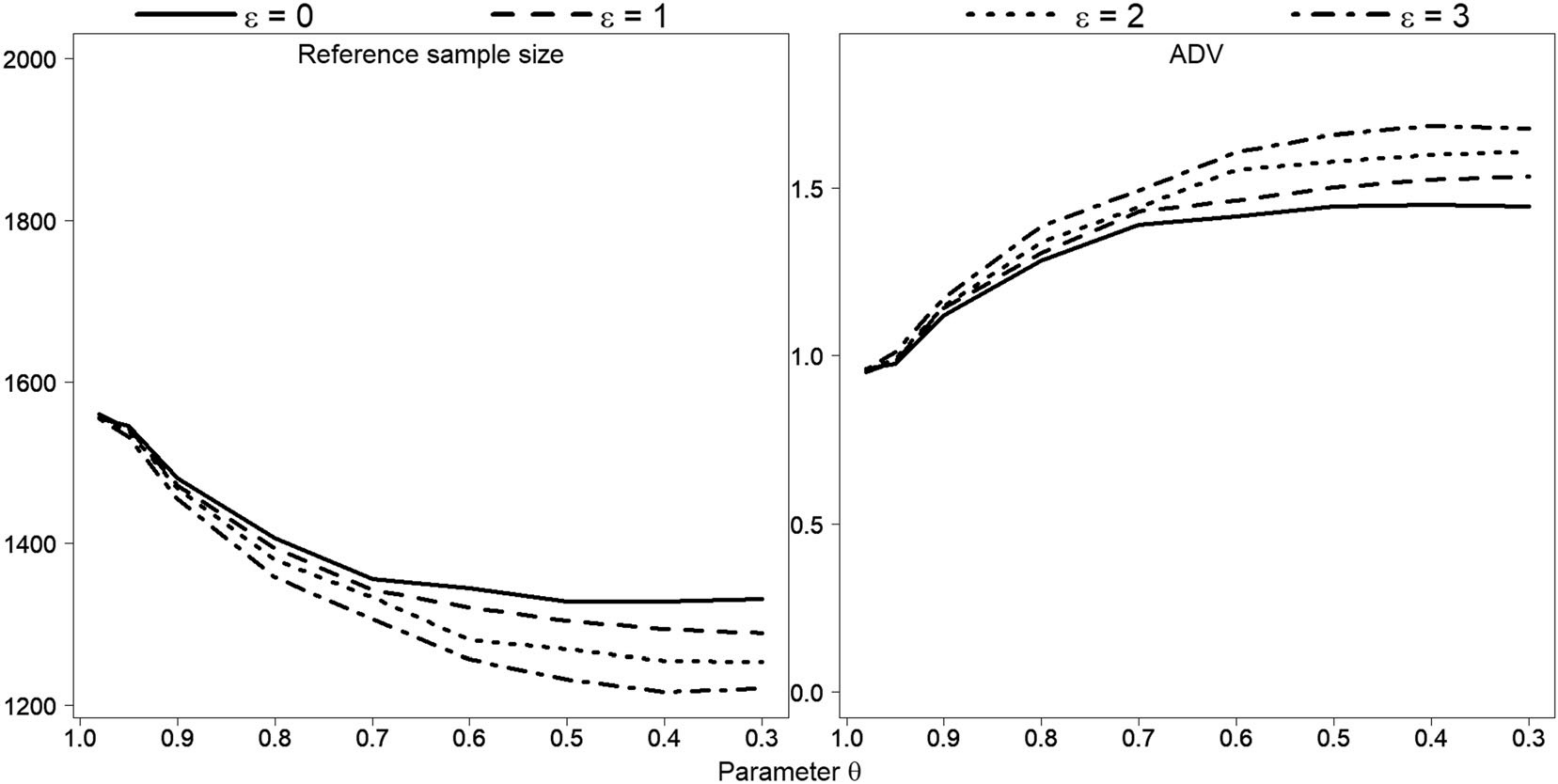
Reference sample size and ADV for various combinations of parameters *ε* and *θ*. Each point represents the average of 1000 simulation runs of data ${\boldsymbol{y}}_{\boldsymbol{i}}\sim \frac{\mathbf{1}}{\mathbf{4}}\ \boldsymbol{N}\left(\mathbf{40},\mathbf{10}\right)+\frac{\mathbf{1}}{\mathbf{2}}\ \boldsymbol{N}\left(\mathbf{70},\mathbf{10}\right)+\frac{\mathbf{1}}{\mathbf{4}}\ \boldsymbol{N}\left(\mathbf{100},\mathbf{10}\right)$**,** ***i*** **= 1,** …**,2000**

In this section, we evaluate the behaviour of the ADV running Monte Carlo simulations. The two parameters *ε* and *θ* explicitly involved in the formula for distance *D* vary across simulations; other parameters are fixed as follows: *γ* = 5, *∆* = 5, and *l*^*I*^ include all length classes. Simulated data for original sample of size 2000 present a superposition of three normal distributions, with means 40, 70 and 100: *y*_*i*_~*π*_1_ *N*(40, *sd*) + *π*_2_ *N*(70, *sd*) + *π*_3_ *N*(100, *sd*), *i* = 1, …, 2000, ${\pi}_1={\pi}_3=\frac{1}{4}$, ${\pi}_2=\frac{1}{2}$. We conduct simulations under standard deviation values *sd* equal to 5, 7 and 10. Thus, for each combination of *ε*, *θ* and *sd*, a set of 1000 replicates are generated (Figs. 14, 15, 16 and 17 illustrate the simulation results).

Taken as a whole, however, our Monte Carlo simulation results confirm the bootstrap results. Under a smaller *sd* value (*sd* = 5), all three modes in the original sample are clearly visible and isolated, so that the entire data can be divided into three well-observed unimodal segments. Therefore, the construction of the reference subsample is rather defined by parameter *θ* than by parameter *ε* (Fig. 8): the lines show almost linear pattern dependent on *θ* but are located close to each other for various *ε* values. For *sd* = 7, the difference between the lines for different *ε* values is clearly more apparent (see Fig. 9). The lines start to flatten out from values *θ* < 0.8, so reference subsample size remains for this values almost unaffected.

For the case of the large *sd* value *sd* = 10, the data (or substantial part of the data) show no apparent pattern, so the modes here are rather weak. Figure 16 illustrates one realization of Monte Carlo simulation for this case, under *θ* = 0.95 and *ε* = 0: the histogram demonstrates a mode in the middle part, but the rest is relative platykurtic. As one can see, the right and left parts of the reference subsample are mostly flat due to the parameter *γ*. Eight hundred fifteen length measurements were already reduced here, but further relaxation in parameter *θ* does not bring any substantial decrease in subsample size, since *γ* affects the amplitude values and blocks the subsampling process.

Figure 17 validates this result: we obtain a significant sample size reduction under larger *θ* values, and then lines are stabilized.

**Fig. 18:**
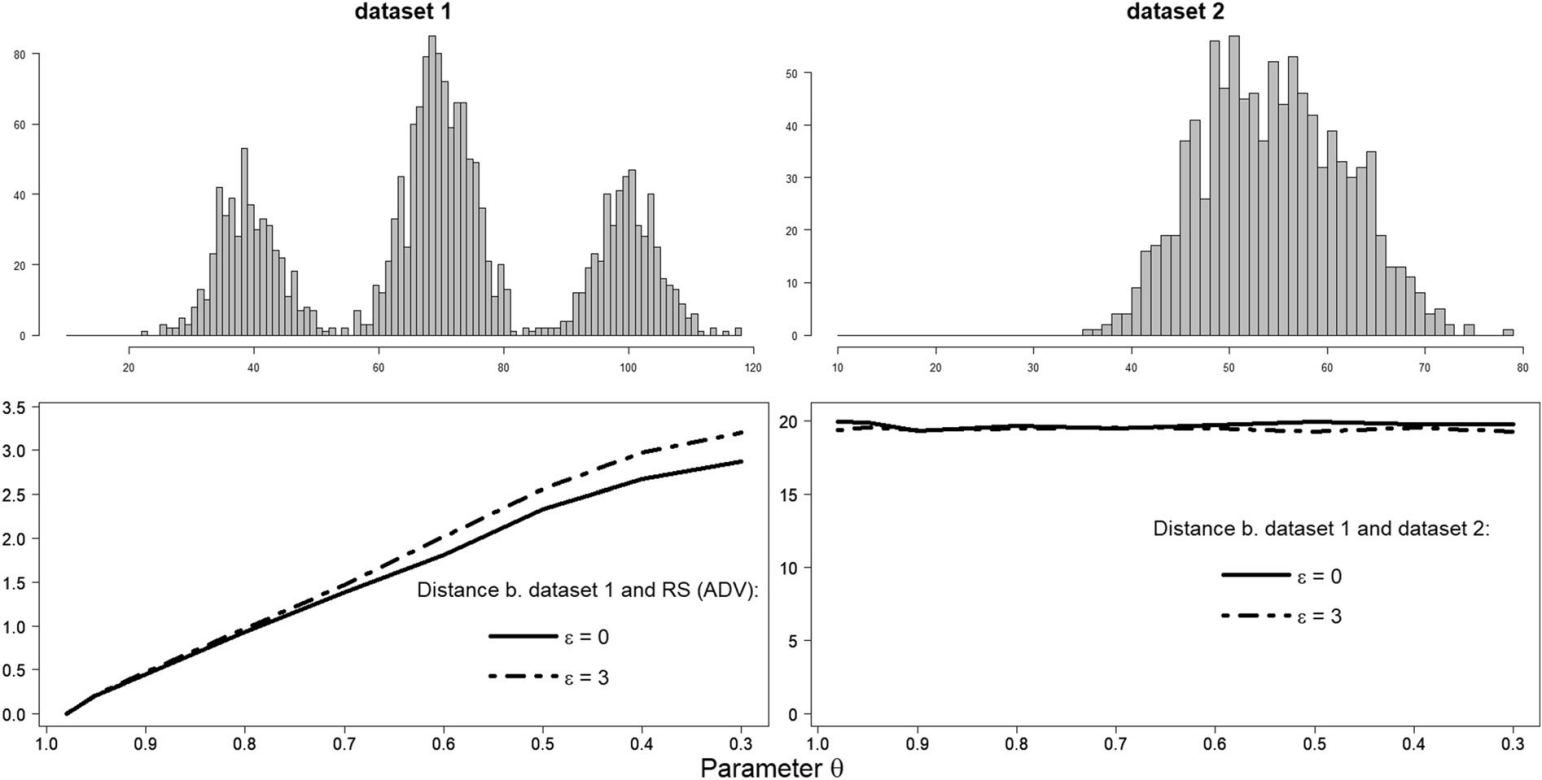
ADV as a dissimilarity indicator of two datasets. Dataset 1: ${\boldsymbol{y}}_{\boldsymbol{i}}\sim \frac{\mathbf{1}}{\mathbf{4}}\ \boldsymbol{N}\left(\mathbf{40},\mathbf{5}\right)+\frac{\mathbf{1}}{\mathbf{2}}\ \boldsymbol{N}\left(\mathbf{70},\mathbf{5}\right)+\frac{\mathbf{1}}{\mathbf{4}}\ \boldsymbol{N}\left(\mathbf{100},\mathbf{5}\right)$, ***i*** **= 1,** …**,2000**. Dataset 2: ${\boldsymbol{z}}_{\boldsymbol{j}}\sim \frac{\mathbf{1}}{\mathbf{2}}\ \boldsymbol{N}\left(\mathbf{50},\mathbf{5}\right)+\frac{\mathbf{1}}{\mathbf{2}}\ \boldsymbol{N}\left(\mathbf{60},\mathbf{5}\right)$, ***j*** **= 1,** …**,1000**

To demonstrate the reliability of ADV, let us consider two simulated datasets (see Fig. 18). The dataset 1 (upper panel left) represents the original sample, for which the reference subsample (RS) was constructed. The ADV plots under *ε* = 0 and *ε* = 3, 0.3 ≤ *θ* < 1, are presented in the lower panel left. The distance values between dataset 1 and dataset 2 (lower panel right) clearly indicate that the dataset 2 (upper panel right) cannot be accepted as a similar one to the dataset 1:

*D*(dataset 1, dataset 2) > > ADV, ∀*θ*, *ε*.

**Fig. 19:**
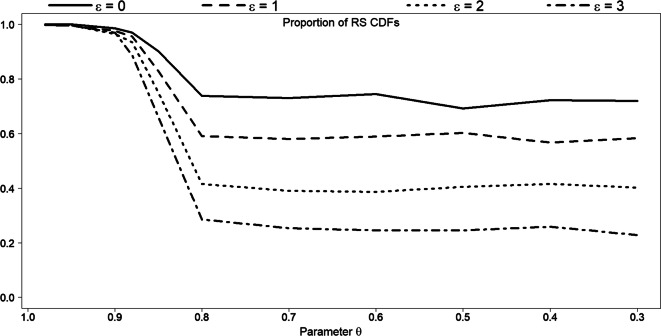
Proportion of reference subsamples CDFs fully enclosed in 95% bootstrap interval of the simulated dataset: ${\boldsymbol{y}}_{\boldsymbol{i}}\sim \frac{\mathbf{1}}{\mathbf{4}}\ \boldsymbol{N}\left(\mathbf{40},\mathbf{7}\right)+\frac{\mathbf{1}}{\mathbf{2}}\ \boldsymbol{N}\left(\mathbf{70},\mathbf{7}\right)+\frac{\mathbf{1}}{\mathbf{4}}\ \boldsymbol{N}\left(\mathbf{100},\mathbf{7}\right)$, ***i*** **= 1,** …**,2000**

Unfortunately, it is difficult to compare our approach to other methods, as our approach is based on Definitions 1 and 2, so does not result in the exact same as, e.g. bootstrap. Still, we have conducted a comparison to the mentioned earlier bootstrap subsampling. However, because of the high computational costs of the bootstrap subsampling, we present a comparison only based on the simulated dataset *S*_0_ employed in Fig. 15: ${y}_i\sim \frac{1}{4}\ N\left(40,7\right)+\frac{1}{2}\ N\left(70,7\right)+\frac{1}{4}\ N\left(100,7\right)$, *i* = 1, …, 2000. As before, we generate 1000 Monte Carlo replicates. For each replicate, we take 500 bootstrap subsamples *S*_*boot*_. Each subsample size is equal to the corresponding mean reference subsample size (see Fig. 15 left). Then, the distance values between subsample and original dataset are determined, and the averaged value over 500 subsamples is calculated. Finally, a grand mean distance value is computed by combining all the individual averages from each replicate. As expected, a direct application of the bootstrap subsampling results in mean distance values, which significantly exceed the corresponding ADVs (in average, *D*(*S*_0_, *S*_boot_) ≈ 14). The empirical CDF of reference subsample, in its turn, not always falls completely within the 95% bootstrap confidence interval constructed for the mean empirical CDF of the original dataset. Figure 19 shows the proportion of reference samples CDFs, entirely enclosed in a 95% bootstrap CI of the original dataset. For 0.8<*θ* < 0.9, the proportion decreases steeply but with slope depending on the *ε* values.

## Discussion

In times of limited resources, a well-designed and quantitatively appropriate sampling scheme for the commercial fishery as an important input to stock assessments is a necessity. Due to limitations in time and staff and partly a lack of willingness by the vessel’s owners to take scientific observers on board, a random sampling of the German commercial fishery is in certain métiers not possible. This leads to a rather opportunistic sampling strategy, taking sampling opportunities when they occur, irrespective if they are planned or not (Ulleweit et al. 2010). Regardless of this drawback, our motivation for developing the presented approach was to optimize the sampling possibilities that we have. Our approach establishes quantitative objectives of length sampling not directly in the form of sample size determination but rather in the form of selection of “information-sufficient”, adequate length distribution.

We defined a “good” representative subsample in both biological and statistical-topological contexts: for instance, despite some moderate difference in central tendency or spread, the distributions of original and subsampled datasets can be quite similar in shapes and therefore reflect biologically the same patterns. Therefore, even if such difference was revealed as statistically significant (e.g. by some statistical test), it might be still too early to draw conclusions. Vice versa, a central tendency in a subsample might remain almost the same, and only large or small lengths may be affected (e.g. due to changes in a sampling scheme) but in a significant way. Many statistical tests will not distinguish between null and alternative hypotheses (Matloff 1991), especially under small or moderate sample sizes. Thus, it has to be measured how different the original and subsampled datasets are, but without relying on the *p* values reported by corresponding statistical tests, bootstrap confidence intervals or selected models—that is, without offering pure statistical approaches to be a major “decision maker”. Our suggestion is that a representative subsample has to match the shape of the original distribution in the main; i.e. capture the important robust distributional patterns of the original sample (or modelled shape), and at the same time ignore fractions which can be just artefacts of insufficient sampling for certain length classes. As example with Kolmogorov-Smirnov test application has shown, the detected difference between original sample and subsample can be real, but just not important, or vice versa. Our approach is aimed to avoid such misinterpretation and helps to conclude with a formal statement regarding the amount of the lost important information in the subsample, with respect to the original sample.

Moreover, the representativeness concept can vary from species to species or can depend on the official national sampling plan. This means that both statistical distributional features and biology experts’ judgements are responsible for reasoning if a subsample is representative and contribute to the decision process. Our framework captures both contributing sides by the choice of the input parameter set {*∆*, *θ*, *l*^*I*^, *ε*}.

The presented effort reducing method results in a suitable combination of trips, hauls and haul fractions, representing a multilevel subsampling scenario, based on the variant (c). This variant illustrates a situation, when (1) the middle part of LFD is of key interest (parameter *l*^*I*^), (2) the magnitudes of the robust critical points of the LFD, probably due to high number of sampling artefacts or measurement inaccuracy (Fig. 2a), get some flexibility to move within the interval defined by parameter *ε*, and (3) the amplitudes between robust critical points can be smoothed (parameter *θ*). Variant (b) includes information also about juveniles in the LDF, and variant (a) might be applied to species with long lifespans, or with large variation in size, so that the tails of distribution are also highly informative and the analysis based on all length classes is preferred. The described flexibility of our approach enables us to apply this method to various fish stocks and fisheries with different selectivity patterns resulting in diverse LDFs in the catches.

The next notes concern potential improvements of the proposed algorithm. First, the setting of the same parameters *θ* and *ε* for all robust critical points $\overrightarrow{V}$ may be too rigorous. As an alternative, we can consider further the vector magnitudes $\overrightarrow{\theta}={\left({\theta}_1,\dots, {\theta}_{\dim \left(\overrightarrow{V}\ \right)-1}\right)}^T$ and $\overrightarrow{\varepsilon}={\left({\varepsilon}_1,\dots, {\varepsilon}_{\dim \left(\overrightarrow{V}\ \right)}\right)}^T$, where each vector component represents the constraints corresponding to the certain critical points. Based on our case study, we could claim that for the major robust mode *V*_1_ = 46 cm as well as for the most obvious robust antimode *V*_2_ = 58 cm, the components of the shift-parameter *ε* are set to *ε*_1_ = *ε*_2_ = 3 cm, but other components give more relaxed constraints, e.g. *ε*_*i* > 2_ = 5 cm. The same concerns amplitude-parameter *θ*: *θ*_1_ = 0.7, but *θ*_*i* > 1_ = 0.5.

We also have to clarify the role of the constants *c*_1_, *c*_2_ and *c*_3_ (see Formal problem statement, dissimilarity measure and admissible dissimilarity value (ADV)). As mentioned above, they are chosen to reflect the relative importance of the constraints (1)-(3) from Definition 2. For instance, our concern was to find only exact matches between LFDs of the original and subsampled datasets with respect to the number of robust critical points, so the large value of *c*_1_ would be appropriate. The constants can be also empirically determined or adapted to the particular LFD content. For example, in the case of slow-growing species, the length classes can overlap considerably, and some of robust modes can appear close to each other. Therefore, if we set here ${c}_2={\left({\min}_{i=1,\dots, \dim \left(\overrightarrow{M}\ \right)}\left|{M}_{i+1}-{M}_i\right|\right)}^{-1}$, a certain softening of the constraint (2) violation for this kind of species will be achieved. Testing the penalty functions and corresponding constants of other types could contribute to future developments of the presented approach.

**Fig. 20:**
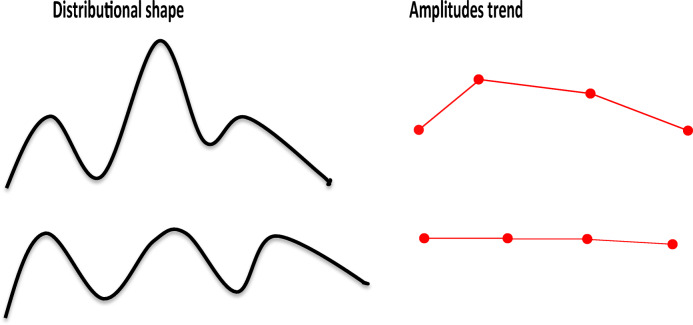
Form of distributional shape and corresponding amplitudes trend

Second, we have to note that Definition 2 should be strengthened, by adding a new condition or improving condition (3). The situation, when the trend of amplitudes between consecutive modes and antimodes can flatten out, is possible. The relative large values (≥ 0.5) of the parameter *θ*, which are also more appropriate in practice, successfully prevent such flattening, but for small *θ*s, this may take place (this situation is explained schematically in Fig. 20). The implication is that both LFD shapes on the left panel can be evaluated as similar, which is clearly faulty: the upper plot is a common one in fisheries sampling multimodal LFD with amplitudes decreasing after the primary mode; the lower one is an “almost sinusoidal” shape with equal amplitudes. The amplitude trends in the right panel verify this. A possible way of modifying the expression for distance *D* could be by entering a penalizing term that, e.g. prevents changes in amplitudes ranks.

Third, combining distance *D*, which is a kind of a shape distribution discriminator, with other metrics like, e.g. coefficient of variation (CV), can improve a performance of the method. Table 2 illustrates an example how mean length and standard error are changed during subsampling. Incorporating an estimation tool for different statistical metrics into the iterative procedure will help to control how the metrics are affected.

Forth, a potential extension of the approach is implementation of a random subsampling procedure, instead of the deterministic one. Currently the use of random subsampling imposes practical complications, like high computational costs and ignoring the important features of the original LFD.

Finally, we have not differentiated between target and incidental catch, as well as between at-sea and onshore sampling, at the current stage of the research. Nevertheless, defining a reference subsample by a set of considered parameters, we can easily extend it or make it more specific. Moreover, the approach is flexible enough to allow the inclusion of parameters or requirements defining the sampling costs and/or environment factors. So, one might be interested in the comparison of costs-precision compromises obtained by elimination of sampled trips or ports (cost savings in on-board maintenance of the observer and salary), hauls (costs and time savings) and measured individuals (rather time savings). In addition, the possibility of multiple species scenario implementation, where practically relevant LFDs for several species are recovered simultaneously, would be a possible topic for future investigations as well. We have focused on length frequency distribution in our approach, but it could also be relevant for distributional analysis of other biological parameters, for example, age. The potential studies are related to the structure of the proposed dissimilarity measure *D* and corresponding parameters we have already highlighted above. The developed framework may be adapted to many possible applications that will be the object of a distinct paper.

Next, we would like to discuss relative advantages and disadvantages of the proposed approach. Previous studies on fish LFDs were based mainly on the implementation of bootstrapping techniques, which definitely can provide a simple solution in problems where analytical solutions do not exist (Efron and Tibshirani 1993). Nevertheless, the application of the bootstrap in fisheries science tasks, apart from computational costs, may be not straightforward. For fisheries data that are clearly not independent and have structure dependence, bootstrap procedures can sometimes become inflexible and hard to modify. An example for some powerful modification might be a multilevel bootstrapping (e.g. Ren et al. 2010; Sturludottir and Stefansson 2019), when resampling is done first on the highest level (e.g. trips) and then sequentially on the lower levels (hauls within each trip, individuals within each haul). Still, as we have already mentioned above, keeping significant distributional patterns in simulated subsamples is not guaranteed here and may lead to the loss of essential patterns. Consequently, the advantages offered by our approach can be described as follows.

1. The approach is based on multiple criteria, which include identification and keeping the significant informative critical points of LFD depending on the species, as well as focusing (if desired) on the “bulk” of the LFD.
2. The approach overcomes the problem of data dependence, because the dependence structure of the original sample is implicitly preserved in the reference subsample. However, if the goal is also the estimation and comparison of parameters such as mean length and CV, we have to include the estimators for parameters of interest used for dependent data in the algorithm, as we have already mentioned above (e.g. the ratio estimator presented by (Pennington and Volstad 1994)). Still, for stock assessment, the precision of length frequency distribution is a primary target.
3. In general, the accuracy of bootstrapping may be poor for small samples. Besides, if the original sample includes important rare extreme values, bootstrapping can undervalue them and thereby exclude some suitable subsamples (scenarios) as well as underestimate the variability in the original sample. Bootstrap as a “shape descriptor” can be also insensitive to some important LFD features, e.g. amplitudes. In our algorithm, we can control requirements to the reference subsamples. The simulated comparison to bootstrap subsampling confirms this. However, we have to note that the bootstrap and presented here approaches do not the exact same things; therefore, the direct comparison is inappropriate. Both approaches rather complement each other.
4. The explicitly formulated definitions of the robust critical points and the statistical topologically and biologically similar samples help to split the original LFD on well-defined length clusters, i.e. “sieves out” the most frequent length classes (modes) as well the boundaries between them (antimodes) and filters out the noise (or measurement artefacts). This makes the approach more pronounced and transparent.
5. The algorithm is computationally cheap and does not require large storage capacity.

A key drawback of the proposed approach is that it requires some expertise about species LFD structure before the algorithm parameters are chosen, and this can include some kind of subjectivity. However, the parameters {*∆*, *θ*, *l*^*I*^, *ε*} define a compromise in length data collection, and this is often practical and/or situational. Therefore, focusing on practical reasons and aspects will minimize the subjectivity. Practical goals, for example, can be guided by regional sampling strategy principles, where the requirements for LFD precision are defined at the regional métier level. The national sampling RDB data should be aggregated and raised to métier level, to determine reference subsamples for species and the corresponding ADVs. So, to achieve a certain level of precision established by these reference subsamples, some redesigns of national sampling strategies might be recommended.

Then, a temporal aspect should be incorporated: e.g. if the elimination of trips made by certain vessel or métier does not affect LFDs presented in our case study for each year, this vessel/métier might be recommended for exclusion from the cod sampling programme. On the other hand, if significant temporal changes in LFD are evident, some additional sampling effort should be allocated to the vessel/métier. Thus, the approach could contribute indirectly to the sampling strategy estimation and can be implemented to find optimal solutions for distributing future sampling tasks in regional sampling programmes. The lack of time component is a clear limitation of the current study. However, this paper is aimed to present the core ideas of the approach. The deeper analysis accounting for a number of years is a conceptual framework of the ongoing research but lies outside the scope of this study.

Our approach relies on identifying (robust) modes/antimodes of LFD. Length data in the original sample, however, not always shows strong cohorts signals, so that LFD shape is nearly uniform or platykurtic and modes/antimodes are not distinct. The subsampling procedure still works and a reference subsample can be obtained, but it might generating misleading conclusions (e.g. identifying one of several small relative equal distributional peaks as a robust mode). In particular, Fig. 17 reflects the simulation results for data partially coming from such a situation. In such cases, the use of methods not relying on modal structure is preferable. Therefore, technically, we would consider the proposed method at its current state as a supporting tool, as a compliment, that can be incorporated as a part into general length-based method processes.

## Conclusion

In this study, we present a novel approach for an optimisation of sampling effort as an alternative to bootstrap and modelling techniques. The length sampling of the German commercial vessels on North Sea cod serves as an example for testing the method and for highlighting the advantages using this approach. Our conclusion is that the approach could be integrated into length-based methods in fisheries science, in order to support the optimization of the sampling process at some stages of national or regional sampling programmes, without reducing the quality of stock assessments. Overall, managers can use it as a complementary tool in the planning and determination future sampling plans and in the evaluation and optimization of existing sampling designs, as well as by fisheries scientists in stock assessments. We suggest that future developments should be focused on developing a more versatile, generalized version of the dissimilarity measure *D* and subsampling algorithm, combined with an application to various sampling designs.
